# *Plasmodium vivax* morbidity after radical cure: A cohort study in Central Vietnam

**DOI:** 10.1371/journal.pmed.1002784

**Published:** 2019-05-17

**Authors:** Thanh Vinh Pham, Hong Van Nguyen, Angel Rosas Aguirre, Van Van Nguyen, Mario A. Cleves, Xa Xuan Nguyen, Thao Thanh Nguyen, Duong Thanh Tran, Hung Xuan Le, Niel Hens, Anna Rosanas-Urgell, Umberto D’Alessandro, Niko Speybroeck, Annette Erhart

**Affiliations:** 1 National Institute of Malariology, Parasitology and Entomology (NIMPE), Hanoi, Vietnam; 2 Research Institute of Health and Society (IRSS), Université Catholique de Louvain (UCL), Brussels, Belgium; 3 Instituto de Medicina Tropical Alexander von Humboldt, Universidad Peruana Cayetano Heredia, Lima, Perú; 4 Fund for Scientific Research (FNRS), Brussels, Belgium; 5 Provincial Health Services, Tam Ky City, Quang Nam Province, Vietnam; 6 Department of Pediatrics, University of Arkansas for Medical Sciences (UAMS), College of Medicine, Little Rock, Arkansas, United States of America; 7 Provincial Malaria Station, Tam Ky City, Quang Nam Province, Vietnam; 8 Center for Statistics, I-BioStat, Hasselt University, Hasselt, Belgium; 9 Centre for health economic research and modelling infectious diseases, Vaxinfectio, University of Antwerp, Antwerp, Belgium; 10 Department of Biomedical Sciences, Institute of Tropical Medicine (ITM), Antwerp, Belgium; 11 Medical Research Council Unit The Gambia (MRCG), the London School of Hygiene and Tropical Medicine, London, United Kingdom; 12 Department of Public Health, ITM, Antwerp, Belgium; Mahidol University, THAILAND

## Abstract

**Background:**

In Vietnam, the importance of vivax malaria relative to falciparum during the past decade has steadily increased to 50%. This, together with the spread of multidrug-resistant *Plasmodium falciparum*, is a major challenge for malaria elimination. A 2-year prospective cohort study to assess *P*. *vivax* morbidity after radical cure treatment and related risk factors was conducted in Central Vietnam.

**Methods and findings:**

The study was implemented between April 2009 and December 2011 in four neighboring villages in a remote forested area of Quang Nam province. *P*. *vivax*-infected patients were treated radically with chloroquine (CQ; 25 mg/kg over 3 days) and primaquine (PQ; 0.5 mg/kg/day for 10 days) and visited monthly (malaria symptoms and blood sampling) for up to 2 years. Time to first vivax recurrence was estimated by Kaplan–Meier survival analysis, and risk factors for first and recurrent infections were identified by Cox regression models. Among the 260 *P*. *vivax* patients (61% males [159/260]; age range 3–60) recruited, 240 completed the 10-day treatment, 223 entered the second month of follow-up, and 219 were followed for at least 12 months. Most individuals (76.78%, 171/223) had recurrent vivax infections identified by molecular methods (polymerase chain reaction [PCR]); in about half of them (55.61%, 124/223), infection was detected by microscopy, and 84 individuals (37.67%) had symptomatic recurrences. Median time to first recurrence by PCR was 118 days (IQR 59–208). The estimated probability of remaining free of recurrence by month 24 was 20.40% (95% CI [14.42; 27.13]) by PCR, 42.52% (95% CI [35.41; 49.44]) by microscopy, and 60.69% (95% CI [53.51; 67.11]) for symptomatic recurrences. The main risk factor for recurrence (first or recurrent) was prior *P*. *falciparum* infection. The main limitations of this study are the age of the results and the absence of a comparator arm, which does not allow estimating the proportion of vivax relapses among recurrent infections.

**Conclusion:**

A substantial number of *P*. *vivax* recurrences, mainly submicroscopic (SM) and asymptomatic, were observed after high-dose PQ treatment (5.0 mg/kg). Prior *P*. *falciparum* infection was an important risk factor for all types of vivax recurrences. Malaria elimination efforts need to address this largely undetected *P*. *vivax* transmission by simultaneously tackling the reservoir of *P*. *falciparum* and *P*. *vivax* infections.

## Abstract 2

In a cohort study in 4 villages in Vietnam, Thanh Vinh Pham and colleagues investigate outcomes 1-2 years following a high dose primaquine regimen to treat P. vivax malaria.

## Author summary

**Why was this study done?:**

- WHO currently recommends a 14-day primaquine (PQ) regimen at 0.5 mg/kg/day together with chloroquine (CQ; 25 mg/kg over 3 days) for the radical cure of *P*. *vivax* infections in Southeast Asia.
- Between 2007 and 2009, Vietnam introduced a high-dose PQ regimen shortened to 10 days in an attempt to improve patient compliance.
- A 2-year one-arm cohort study was conducted between May 2009 and December 2011 in four remote villages of Central Vietnam to determine the incidence and risk factors for vivax recurrence after the new radical cure regimen.

**What did the researchers do and find?:**

- 260 *P*. *vivax*-infected patients were enrolled between May 2009 and December 2010, received directly observed 10-day treatment, and were followed up monthly for up to 24 months.
- During scheduled and unscheduled visits, participants were examined, and blood samples were systematically taken for light microscopy (LM) and polymerase chain reaction (PCR) analysis. All LM-detected vivax infections (patent) were treated with CQ alone, while PQ was given at the end of individual follow-up.
- Time-to-event analysis showed that by month 24, only 20% of patients remained free of vivax recurrences detected by PCR, while this was 40% for patent (by LM) recurrences. The majority of recurrences were submicroscopic and asymptomatic because only 38% of patients had symptomatic recurrences.
- The main risk factors for *P*. *vivax* recurrences were prior *P*. *falciparum* infections, especially submicroscopic, and prior treatment of vivax infections with CQ.

**What do these findings mean?:**

- Despite the age (10 years) of the data collected, the results of this cohort study remain relevant to the current malaria situation in Vietnam, where the elimination of vivax malaria and of multidrug-resistant *P*. *falciparum* are major challenges.
- A substantial proportion of the vivax malaria reservoir is not detected by current surveillance strategies in Vietnam, i.e., passive and re-active case detection based on LM/rapid diagnostic tests (RDTs).
- Prior *P*. *falciparum* infections (including submicroscopic) and vivax infections treated with CQ alone were significant risk factors for vivax recurrences.
- To further improve vivax elimination efforts in Vietnam, new treatment strategies (i.e., single-dose radical treatment) and more sensitive diagnostic tools (i.e., field PCR/ultrasensitive RDTs) simultaneously tackling falciparum and vivax malaria infections are needed.

## Introduction

Radical cure of *P*. *vivax* malaria, the most prevalent species outside sub-Saharan Africa [1], remains a challenge because preventing subsequent relapses and onward transmission requires treatment of both circulating blood (schizonticidal drugs) and the dormant liver (hypnozoiticidal drug) stages [2]. Chloroquine (CQ) and primaquine (PQ) have been combined for this purpose since the late 1950s, and PQ—an 8-aminoquinoline licensed in 1952—remained the only antirelapse therapy available until July 2018 [3]. Its use implies several challenges, including the risk of acute hemolysis in Glucose-6-Phosphate-dehydrogrenase–deficient (G6PDd) patients and poor adherence to the 14-day treatment schedule [2]. Moreover, the rapid spread of *P*. *vivax* CQ resistance will require alternative blood schizonticides and thus new combinations for radical cure to be tested [4]. Over the past 60 years, various PQ regimens have been used [5], and currently the World Health Organization (WHO) guidelines recommend PQ at 0.25 mg/kg/day for 14 days, together with CQ or an artemisinin-based combination therapy (ACT) [6]. In Southeast Asia and Oceania, where the vivax Chesson strain is prevalent, a higher PQ daily dose (0.5 mg/kg/day; 7.0 mg/kg total dose) is recommended.

In Vietnam, the importance of *P*. *vivax* malaria has recently increased following the successful control efforts against *P*. *falciparum* [7] and the recent country’s commitment to eliminate malaria by 2030 [8]. Currently, malaria transmission remains confined in forested areas of Central Vietnam, with the highest incidence reported along the international borders with Laos and Cambodia [9]. Between 2011 and 2015, the annual incidence of confirmed malaria cases dropped by 44% (i.e., from 16,612 to 9,331) and malaria deaths by 79% (3 deaths recorded in 2015). Concomitantly, the annual incidence of confirmed *P*. *falciparum* cases decreased by 57% (from 10,101 to 4,327 cases) while *P*. *vivax* cases decreased only by 15% (from 5,602 to 4,756 cases). Consequently, the frequency of *P*. *vivax* relative to *P*. *falciparum* increased from 34% to 51%, a significant change because *P*. *vivax* consistently accounted for 20%–30% of all malaria cases since 1991 [10].

In the framework of the current National Malaria Control and Elimination Program (NMCEP) launched in 2011, surveillance strategies include both passive case detection (PCD) using light microscopy (LM) or rapid diagnostic tests (RDTs) (at the health centers, hospitals, and private clinics) and re-active case detection (Re-ACD) following the detection of any malaria case (“index case”) by PCD at community level [11]. Re-ACD consists of screening all residents within 20–30 houses around the index case for malaria (using RDTs/LM) and treating all positive individuals. Re-ACD is done by a mobile team (including health staff and Hamlet Health Workers [HHWs]) as part of the “2-4-7 surveillance and response strategy” adapted from China [12]. All antimalarial treatments are provided free of charge by the NMCEP, and the current guidelines for the radical treatment of *P*. *vivax* recommend the use of PQ at 0.25 mg/kg/day for 14 days together with CQ for 3 days [13].

However, over the past 20 years, treatment guidelines for vivax malaria in Vietnam have changed several times, illustrating the difficult balance between maximizing the effectiveness of and compliance to PQ while minimizing the risk of acute hemolysis in G6PDd patients. Therefore, after the 5-day PQ regimen at 0.5 mg/kg/day introduced in 1997, guidelines were changed to 0.25 mg/kg/day for 10 days in 2003 and to 0.50 mg/kg/day for 10 days between 2007 and 2009, the latter of which was used in the present study [14]. However, in 2009, this high-dose regimen was again changed to PQ at 0.25 mg/kg/day for 14 days [15] following increased fear of hemolysis in the absence of readily available G6PD testing. In remote areas of Central Vietnam, where treatment monitoring is challenging, adherence to PQ treatment is reportedly low because of both fear by the health staff of acute hemolysis in G6PDd patients and poor patient compliance to the 14-day course [16]. These challenges related to PQ treatment are commonly reported in all vivax-endemic areas [17,18].

We report here the results of a cohort study in which *P*. *vivax* patients were treated radically with the previously recommended high-dose PQ regimen (0.50 mg/kg/day for 10 days) and followed up monthly for up to 2 years, with the aim of evaluating the post-treatment transmission dynamics of recurrent vivax infections.

## Materials and methods

Data were collected following a prospective study protocol that did not include a detailed prospective statistical analysis plan (S1 Text).

### Study design

This is a 2-year prospective cohort study carried out in Central Vietnam in which *P*. *vivax*-infected patients were followed up monthly after radical treatment. At each visit, a clinical examination and blood sampling for malaria parasite identification by LM and polymerase chain reaction (PCR) were performed. During the follow-up, vivax recurrent infections identified by LM and/or symptomatic infections were treated with CQ alone. This aimed at better determining the vivax parasite reservoir in real-life conditions because PQ was not usually administered to these patients at the time this project was implemented.

### Study site and population

The study was implemented between April 2009 and December 2011 in four neighboring villages (Villages 1–3 in the Tra Leng commune and Village 4 in the Tra Don commune) in the mountainous and forested district of Nam Tra My in Quang Nam province. Villages were organized in several scattered clusters of about 4 to 45 households each (total population of 1,810 individuals according to the March 2009 census). A detailed description of the sociodemographic characteristics and malariometric indices of this study area has been published elsewhere [19]. Briefly, the study population belonged to the M’nong (Villages 1–3) and Cadong (Village 4) ethnic groups, living mainly off slash-and-burn agriculture and cinnamon plantations in forest fields. Malaria transmission is perennial with two peaks, May–June and October–November, with two main vector species: *Anopheles dirus sensu stricto* and *A*. *minimus sensu stricto* [20,21]. In April 2009, at baseline, malaria prevalence in the four study villages was 7.8% by LM and 23.6% by PCR, with 60% *P*. *falciparum* and the remaining *P*. *vivax* infections [19]. The G6PD genetic polymorphism (Vianchiang mutation) was estimated at less than 1.5% in both males and females, with no difference between ethnic groups.

### Data collection

*P*. *vivax*-infected patients were first identified during the baseline survey (April–May 2009) [19] and then by PCD at the Tra Leng Community Health Center (CHC) (May 2009–Dec 2010) until the target sample size was reached (see below). Inclusion criteria were age ≥3 years (following contemporary guidelines [14]; changed to ≥6 months in the current guidelines [15]) and ≤60 years old, axillary temperature ≥37.5 °C and/or history of fever in the previous 48 hours, microscopically confirmed *P*. *vivax* monoinfection (asexual stage), permanent residency in the study area, and ability and willingness to participate in the study confirmed by a written informed consent (parents/guardians for patients <18 years). Patients were excluded if they had any danger signs or severe malaria, any concurrent infection or underlying chronic condition (e.g., tuberculosis [TB], HIV, epilepsy, etc.) requiring specific treatment, severe malnutrition, known allergy or intolerance to study drugs, prior PQ treatment within the past month, or if they were pregnant (as confirmed by rapid test) or breastfeeding. Patients were treated daily with CQ (25 mg/kg over 3 days) and PQ (0.5 mg/kg/day for 10 days) according to the contemporary national guidelines [15]. During the first 10 days of follow-up, patients were examined daily, and treatment was directly observed. Patients were then asked to attend the CHC at day 14, 21, and 28 or if ill between scheduled visits. Any patent vivax recurrence was considered as treatment failure; patients received rescue treatment (dihydroartemisinin-piperaquine [DHA-PQ]) and PQ (10 days), and follow-up was stopped. From day 28 onwards, monthly home visits were done either until completing 2-year follow-up or the end of study in December 2011. Between scheduled visits, patients were advised to consult their HHW or the study team at the CHC if unwell. At each visit (scheduled or unscheduled), they were systematically interviewed, body temperature was collected, and a finger prick blood sample was taken for LM (thick and thin film) and later molecular analysis (filter paper blood sample [FPBS]). Any LM-confirmed *P*. *vivax* infection identified during the monthly follow-up was treated with a 3-day course of CQ; PQ was given to all patients at the end of the follow-up. In addition, LM-confirmed *P*. *falciparum* infections (either mono- or mixed infections) were treated with a 3-day course DHA-PQ as per national guidelines. All patients were closely followed up by the study team for the detection of adverse events, which were managed as per study protocol.

### Definitions

The main study outcomes were the incidence and risk factors for vivax recurrence as measured by time-to-event analysis. A recurrence was defined as a vivax infection detected after the initial post-treatment parasite clearance. Because the parasite reservoir in low-transmission settings is mostly represented by submicroscopic (SM) and asymptomatic infections [19,22], vivax recurrences were systematically detected by both PCR (Seminested Multiplex [SnM] PCR detecting densities below 1 parasite/μl [23,24]) and LM (detection limit of 10–50 parasites/μl for experienced technicians). Therefore, three outcome variables were defined as follows: i) all recurrences identified by PCR (= “all PCR-detected recurrences”); ii) all recurrences identified by PCR and LM (= “patent recurrences”); and iii) all patent recurrences with malaria symptoms (i.e., body temperature ≥37.5 °C and/or other symptoms; = “symptomatic recurrences”).

The following covariates were considered for the risk factor analysis: village, ethnic groups (M’nong, Ca Dong), gender, age, occupation (farmers, other), bed net in house (none, at least one), economic level (low, medium, high), year of recruitment (2009, 2010), season of recruitment (dry, rainy), parasite density at day 0 (parasite/μl), and “prior *P*. *falciparum* infection within 2 months before *P*. *vivax* recurrence” (i.e., all PCR-detected *P*. *falciparum* infections). In addition, we also explored whether being treated with an antimalarial (ACT or CQ) during the previous visit was associated with a higher risk of *P*. *vivax* recurrence. Among risk factors, the economic level was computed using three different variables for livestock ownership, i.e., number of i) buffaloes, ii) cows, and iii) pigs, and by using principal component analysis as described previously [19].

### Laboratory procedures

Parasite species and density were determined by LM. Thin films were fixed with methanol for 15–30 seconds; both thin and thick smears were stained with 3% Giemsa solution for 45 minutes. *P*. *vivax* and *P*. *falciparum* asexual stages were counted against 200 white blood cells (WBCs), assuming a mean WBC count of 8,000/μl. Quality control was done at the National Institute of Malariology, Parasitology, and Entomology (NIMPE), Hanoi, Vietnam by a senior technician blinded to patients’ details. All positive blood slides and 10% of randomly chosen negative slides were double-checked; in case of discrepancy, slides were read by a third senior technician.

FPBSs were first stored at 4 °C until transferred to NIMPE where they were stored at −20 °C until processing. DNA extraction was done using the QIAamp DNA Micro Kit (Qiagen, Hilden Germany); a species-specific SnM-PCR to detect the four human *Plasmodium* species was performed [25]. Quality control was done at the Institute of Tropical Medicine, Antwerp (ITMA) by a senior technician who blindly reread 10% of randomly chosen blood samples.

### Sample size

The sample size was calculated on the basis of *P*. *vivax* CQ failure rates in different sentinel sites in Central Vietnam, ranging from 0% to 5.7% [26]. Assuming 5% treatment failure at day 28 post-treatment and 10% loss to follow-up, 205 patients would provide 3% precision at a 5% significance level (“CSample” command/Epi Info6). Beyond 28 days post-treatment, it was assumed that 20% of the remaining patients would be lost to follow-up. Therefore, an additional security margin was added to start with a cohort of 250 patients, resulting in 205 patients with complete follow-up data points (i.e., D0 + 24 monthly records).

### Data analysis

Data were double entered and checked in Epidata version 3.1 (The EpiData Association, Odense M, Denmark), and analyzed using STATA version 11 (Stata Corp, College Station, TX, USA). The study design was taken into account at all steps of the analysis by setting the data with household as primary sampling unit and village as strata (alpha = 0.05) using the survey (“*svy*”) command in STATA.

All patients entered the follow-up at D0 until censoring, which occurred when patients were lost follow-up, had a treatment failure during the first 28 days, or at the end of the monthly follow-up. Any patent recurrence after day 28 was systematically treated with CQ (3 days) and considered as an independent event from the next one. However, the SM recurrences that could be detected for several consecutive months in the same individual (but were not treated because they were identified months later) were considered as “positive PCR person-month.”

Summary statistics (accounting for study design) were used to describe the baseline characteristics at inclusion and at subsequent visits. Incidence rate of vivax recurrences were calculated by dividing the number of recurrent episodes by the person-years at risk and expressed as the number of recurrences per person-years at risk. The Wilcoxon sign rank test was used to compare the median time intervals between consecutive recurrences identified by PCR.

Time to first *P*. *vivax* recurrence post-treatment was analyzed by Kaplan–Meier survival analysis, which estimated the probability for patients to remain free of any vivax recurrence (PCR-detected, patent, or symptomatic) as well as of any *P*. *falciparum* infection (detected by PCR). To minimize the risk of vivax infections acquired outside the study area, individuals absent from the study area for two or more consecutive visits were censored at their last visit. In case of a single monthly visit missed, this was not considered as a missing month if the individual had remained within the study site, while if s/he had left the study area, it was considered as a person-time at risk censored at the time s/he was absent.

The time at risk to first recurrence was counted as days from D0 to first vivax recurrence, while for any subsequent recurrence, the time at risk was counted as days since the previous recurrence. Patients treated with either CQ (patent *P*. *vivax* recurrence) or DHA-PQ (patent *P*. *falciparum* infection) were censored for 14 days post-treatment.

The risk factor analysis was conducted both for the first and recurrent vivax recurrences. A uni- and multivariable-adjusted Cox Proportional Hazard (PH) regression model (Hazard Ratio [HR]) was used to identify significant risk factors for the first PCR-detected *P*. *vivax* recurrence (first recurrence per subject). A Cox Conditional Gap Time (CGT) model was used to assess the risk for recurrent infections per subject over time [27,28]. In this model, each recurrence was analyzed separately and stratified by recurrence order, the underlying assumption being that a subject was not at risk of a second recurrence until the first recurrence had occurred. Thus, the conditional risk set at time for recurrence *k* (*t*_*k*_) was made up for all subjects under observation at time they had had recurrence *k* − 1. This method considers the total number of recurrences analyzed and the time at risk between each recurrence. The extra correlation due to repeated observations within patients was accounted for (using the *vce* (*cluster ID*) option within the “*stcox*” command in the CGT model). Interactions were systematically checked up to order two. The PH assumption was tested for each Cox model.

### Ethical clearance

Ethical clearance was obtained from the ethical committees of both the NIMPE, Hanoi and the University of Antwerp. The fundamental principles of ethics in research on human participants were upheld throughout the project. The study objectives and methods were first explained to the community leaders for their approval. Each study participant provided a written informed consent following explanation of the study objectives and follow-up procedures as well as their right to withdraw without prejudice for themselves or their families. For minors (<18 years old), parents/guardians signed the individual informed consent, while individuals aged 12–18 were asked to provide individual assent.

## Results

### Cohort characteristics

**Fig 1:**
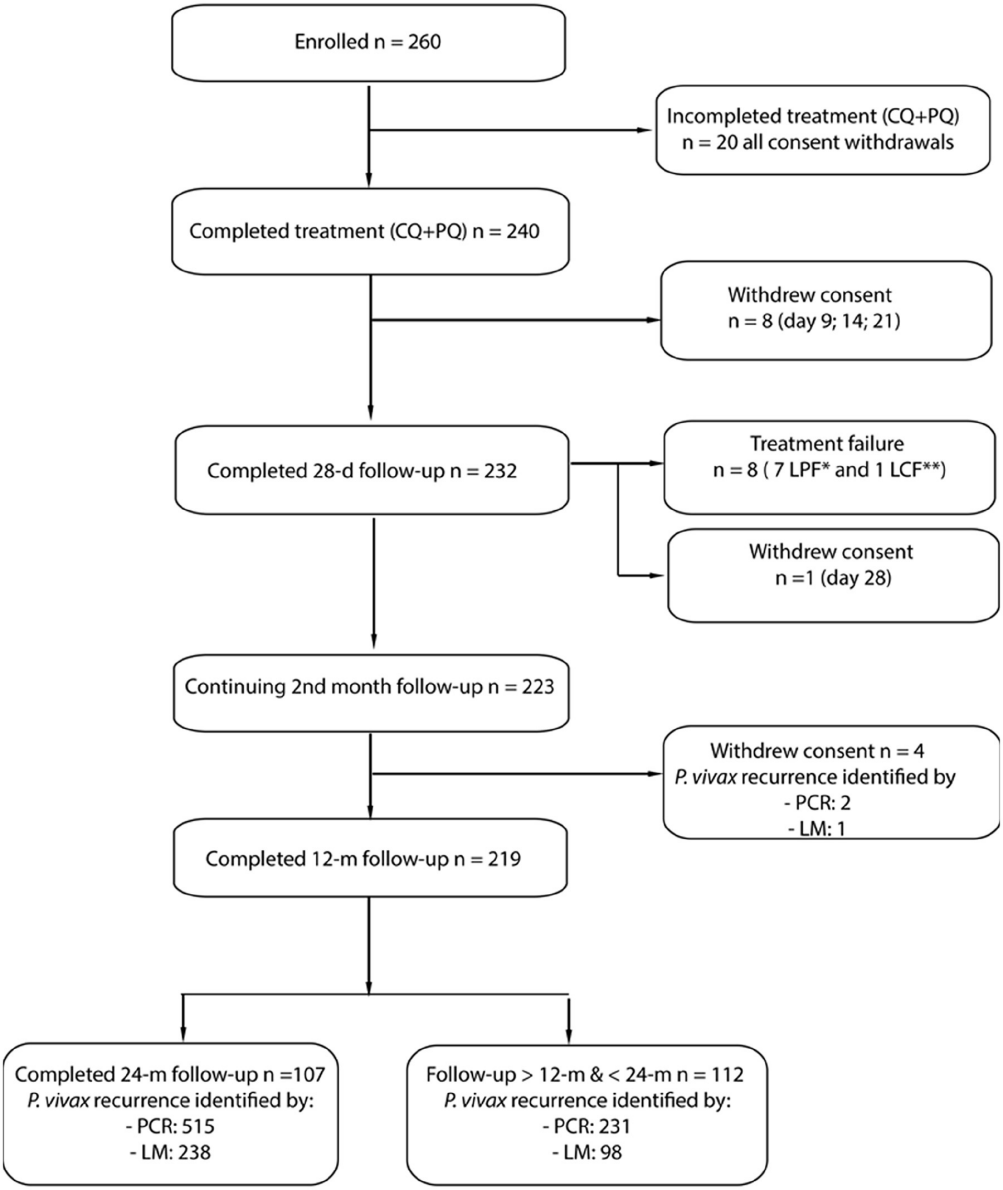
Study profile. CQ, chloroquine; d, day; LCF, Late Clinical Failure; LM, light microscopy; LPF, Late Parasitological Failure; m, month; PCR, polymerase chain reaction; PQ, primaquine.

A total of 260 *P*. *vivax*-infected patients were identified and enrolled (21 during initial survey; 239 during PCD). Of these, 20 (7.69%) patients withdrew consent and did not complete the 10-day radical cure (Fig 1). By day 28 (first monthly visit), 9 additional patients had withdrawn consent, and 8 had treatment failure, were treated with DHA-PQ (3 days) and PQ (10 days), and were excluded as per protocol. Therefore, after day 28, the cohort comprised 223 patients, of whom 4 withdrew consent (moved out of study area), 219 completed at least 12 months, and 107 completed 24 months of follow-up. Overall, 33 patients (33/260 = 12.7%) withdrew consent, mainly (20/33 = 60.6%) during the 10-day PQ treatment. Total individual follow-up times ranged from 9 to 719 days, with a median of 628 days (IQR 508–718), and none of the participants were censored because of prolonged absence outside the study area.

Most patients were males (sex ratio M/F = 1.6), and children 3–9 years old represented almost half (43.08%) of the cohort (Table 1). The M’nong ethnic group (78.46%) in Villages 1–3 was the most represented. All adults were farmers. Most patients were very poor (lowest income category = 56.54%) and without a bed net at home (70.77%). Beside fever and/or history of fever, the most frequent symptoms at enrollment were headache and fatigue. The spleen rate was 6.15% (16/260). The mean parasite density at enrollment was 2,754.1/μl, and gametocytes were found in most infections (86.15%). By day 3, all patients had cleared infection and were symptom-free. About half of the patients were recruited in 2009 (48.46%), and the rest in 2010. The sociodemographic characteristics of patients recruited in 2009 were similar to those recruited in 2010 except for village, ethnicity, and bed net availability (S1 Table).

**Table 1:** Baseline characteristics of the 260 patients enrolled in the cohort.

Variables	*n*	%	95% CI
**Village**			
Village 1	101	38.85	[34.83; 43.02]
Village 2	64	24.62	[21.25; 28.33]
Village 3	39	15.00	[12.84; 17.45]
Village 4	56	21.54	[17.63; 26.04]
**Gender**			
Male	159	61.15	[55.76; 66.82]
**Ethnic groups**			
M′nong	204	78.46	[73.96; 82.37]
Cadong	56	21.54	[17.63; 26.04]
**Age groups**			
3–9	112	43.08	[37.45; 48.89]
10–19	71	27.31	[21.84; 33.55]
20–29	44	16.92	[12.75; 22.11]
30–60	33	12.69	[9.35; 17.00]
**Occupation**			
No (children <6 years old)	70	26.92	[22.02; 32.47]
Farmer	85	32.69	[27.51; 38.34]
Pupil	105	40.38	[34.34; 46.74]
**Bed net in house**			
No	184	70.77	[61.81; 78.37]
At least one	76	29.23	[21.63; 38.19]
**Economic status**			
Lowest income	147	56.54	[47.20; 65.43]
Low	26	10.00	[5.70; 16.96]
Higher	87	33.46	[25.18; 42.90]
**Year of recruitment**			
2009	126	48.46	[41.80; 55.18]
2010	134	51.54	[44.82; 58.20]
**Parasite density at day 0** (per μl)			
≤1,000	56	21.54	[16.90; 27.03]
1,001–5,000	104	40	[33.61; 46.75]
>5,001	100	38.46	[31.86; 45.52]
Asexual parasites/μl (geometric mean)	2,754.07		[2,271.87; 3,338.61]
**Infections with gametocytes**	224	86.15	[81.37; 89.86]
**Clinical symptoms**			
Fever (axillary temperature ≥37.5 °C)	154	59.23	[53.06; 65.12]
Headache	94	36.15	[30.42; 42.32]
Fatigue	86	33.08	[27.23; 39.50]
Dizziness	28	10.77	[7.51; 15.22]
Enlarged spleen	16	6.15	[3.58; 10.39]
No symptoms	66	25.38	[20.36; 31.16]

The score into tertiles was defined as “high,” “medium,” and “low” economic status.

### Characteristics of vivax recurrences

About two-thirds (76.68%, 171/223) of individuals had at least one PCR-detected recurrence (median 4 recurrences per patient [IQR 2–6]), 124 (55.61%) at least one patent recurrence (median = 2/patient; IQR 1–4), and 84 (37.67%) at least one symptomatic recurrence (median = 1; IQR 1–2) (Table 2). Most patients with PCR-detected (78.95%) and patent recurrences (65.32%) had multiple events, while those with symptomatic recurrences (63.10%) experienced mostly one event. Among the 4,604 visits during the study period, 71 (1.54%) were unscheduled because of symptomatic vivax recurrences.

**Table 2:** Characteristic of *P*. *vivax* recurrences (all PCR-detected, patent, and symptomatic) detected between May 2009 and December 2011.

*P*. *vivax* recurrences	All PCR-detected		Patent		Symptomatic	
	*n*	%	*n*	%	*n*	%
Patients free of recurrence	52	23.32	99	44.39	139	62.33
**Patients with recurrence:**	**171**	**76.68**	**124**	**55.61**	**84**	**37.67**
1 recurrence only	36	21.05	43	34.68	53	63.10
2 recurrences	22	12.87	25	20.16	19	22.62
3 recurrences	20	11.70	18	14.52	6	7.14
4+ recurrences	93	54.38	38	30.64	6	7.14
Number of recurrences/individual (Median [IQR]; max)	4 [2–6]; 13		2 [1–4]; 10		1 [1–2]; 7	
Median time from enrollment to first recurrence (days), [IQR]	118 [59–208]		141 [86–237]		181 [97–316]	
**Time intervals from enrollment to first recurrence:**						
By day 28	5	2.93	0	0.00	0	0.00
29–180 days	113	66.08	80	64.52	42	50.00
>180 days	53	30.99	44	35.48	42	50.00

**Abbreviations**: IQR, Interquartile Range; PCR, polymerase chain reaction.

Among the 748 PCR-detected *P*. *vivax* recurrences, 45% (337/748) were patent, and among these, 41% (139/337) were symptomatic. The incidence rate was 1.98 per person-years (95% CI [1.84; 2.12]) for PCR-detected, 0.89 per person-years (95% CI [0.80; 0.99]) for patent, and 0.37 per person-years (95% CI [0.31; 0.43]) for symptomatic recurrences (Table 3). Additionally, a total of 285 *P*. *falciparum* infections were detected by PCR (median: 2 episodes/person [IQR 1–3]; maximum 8/person) of which 240 (84.21%) were patent (median: 1 episode/person [IQR 1–3]; maximum 8), including 149 (/240 = 62.08%) symptomatic infections (median = 1/person [IQR 1–2]; maximum 6).

**Table 3:** Univariable risk factor analysis for first recurrence using Cox PH model and for all recurrences using Cox CGT model (*n* = 223).

Risk factor		Univariate of first recurrence					Univariate of all recurrences				
		*n*/per year	IR	HR	*P*-value	95% CI	*n*/per year	IR	HR	*P*-value	95% CI
**All PCR-detected recurrences**	**Overall**	171/148.64	1.15			[0.99; 1.34]	748/378.38	1.98			[1.84; 2.12]
	**Village**										
	Village 1	68/76.40	0.89	1			288/161.07	1.79	1		
	Village 2	40/34.48	1.16	1.18	0.479	[0.74; 1.87]	144/82.54	1.74	0.97	0.811	[0.79; 1.20]
	Village 3	22/24.33	0.9	0.91	0.705	[0.56; 1.48]	74/53.33	1.39	0.83	0.205	[0.61; 1.11]
	Village 4	41/13.43	3.05	3.02	<0.001	[2.09; 4.36]	242/81.43	2.97	1.27	0.007	[1.07; 1.51]
	**Ethnic group**										
	M’nong	130/135.21	0.96	1			506/296.95	1.70	1		
	Cadong	41/13.43	3.05	2.92	<0.001	[2.06; 4.14]	242/81.43	2.97	1.32	<0.001	[1.13; 1.53]
	**Prior *P*. *falciparum* infection before *P*. *vivax* recurrence**										
	No	154/147.09	1.05	1			645/369.41	1.75	1		
	Yes	17/1.55	10.94	6.82	<0.001	[5.13; 9.06]	103/8.96	11.49	4.90	<0.001	[4.19; 5.73]
	**Year of recruitment**										
	2009	99/63.39	1.56	1			526/212.85	2.47	1		
	2010	72/85.25	0.84	0.57	0.001	[0.42; 0.79]	222/165.53	1.34	0.69	<0.001	[0.57; 0.82]
	**Bed net in house**										
	No	130/95.02	1.37	1			579/272.22	2.13	1		
	At least one	41/53.62	0.76	0.59	0.011	[0.39; 0.88]	169/106.16	1.59	0.86	0.119	[0.72; 1.04]
	**Prior ACT/CQ treatment before *P*. *vivax* recurrence**										
	No						653/368.66	1.77	1		
	Yes						95/9.72	9.78	5.08	<0.001	[4.32; 5.96]
	***P*. *vivax* density/μl at day 0**										
	<1,000	37/31.42	1.18	1			204/86.50	2.36	1		
	1,000–5,000	62/55.71	1.11	0.93	0.721	[0.62; 1.40]	252/138.44	1.82	0.83	0.043	[0.69; 0.99]
	>5,000	72/61.52	1.17	0.97	0.887	[0.66; 1.43]	292/153.44	1.90	0.85	0.070	[0.71; 1.01]
	**Economic status**										
	Very low income	99/83.90	1.18	1			385/216.37	1.78	1		
	Low	16/15.00	1.07	0.90	0.72	[0.49; 1.64]	66/35.14	1.88	1.01	0.926	[0.80; 1.27]
	Higher	56/49.75	1.13	0.95	0.762	[0.66; 1.36]	297/126.87	2.34	1.21	0.015	[1.04; 1.40]
**Patent recurrences**	**Overall**	124/213.09	0.58			[0.49; 0.69]	337/378.38	0.89			[0.80; 0.99]
	**Village**										
	Village 1	52/96.47	0.54	1			124/161.07	0.77	1		
	Village 2	24/54.35	0.44	0.79	0.432	[0.45; 1.41]	53/82.54	0.64	0.82	0.310	[0.56; 1.20]
	Village 3	11/37.85	0.29	0.57	0.068	[0.31; 1.04]	31/53.33	0.58	0.84	0.468	[0.53; 1.34]
	Village 4	37/24.42	1.52	2.53	<0.001	[1.68; 3.81]	129/81.43	1.58	1.59	<0.001	[1.23; 2.07]
	**Ethnic group**										
	M’nong	87/188.67	0.46	1			208/296.95	0.70	1		
	Cadong	37/24.42	1.52	2.95	<0.001	[2.00; 4.35]	129/81.43	1.58	1.72	<0.001	[1.36; 2.18]
	**Prior *P*. *falciparum* infection before *P*. *vivax* recurrence**										
	No	107/210.42	0.51	1			280/369.41	0.76	1		
	Yes	17/2.67	6.38	7.97	<0.001	[5.57; 11.40]	57/8.96	6.36	5.16	<0.001	[3.93; 6.77]
	**Year of recruitment**										
	2009	82/97.36	0.84	1			244/212.85	1.15	1		
	2010	42/115.73	0.36	0.43	<0.001	[0.28; 0.65]	93/165.53	0.56	0.59	0.003	[0.42; 0.83]
	**Bed net in house**										
	No	96/142.67	0.67	1			271/272.22	1.00	1		
	At least one	28/70.42	0.40	0.61	0.032	[0.39; 0.96]	66/106.16	0.62	0.72	0.038	[0.53; 0.98]
	**Prior ACT/CQ treatment before *P*. *vivax* recurrence**										
	No						290/368.66	0.79	1		
	Yes						47/9.72	4.84	6.39	<0.001	[4.62; 8.83]
**Symptomatic recurrences**	**Overall**	84/227.14	0.31			[0.25; 0.28]	139/378.38	0.37			[0.31; 0.43]
	**Village**										
	Village 1	37/118.33	0.31	1			51/161.07	0.32	1		
	Village 2	11/69.79	0.16	0.48	0.036	[0.24; 0.95]	14/82.54	0.17	0.53	0.039	[0.29; 0.97]
	Village 3	10/40.72	0.25	0.78	0.475	[0.40; 1.54]	17/53.33	0.32	1.02	0.946	[0.55; 1.88]
	Village 4	26/43.30	0.61	2.02	0.022	[1.11; 3.69]	57/81.43	0.70	2.09	<0.001	[1.38; 3.15]
	**Ethnic group**										
	M’nong	58/228.84	0.25	1			82/296.95	0.28	1		
	Cadong	26/43.30	0.60	2.53	0.001	[1.45; 4.41]	57/81.43	0.70	2.36	<0.001	[1.62; 3.45]
	**Prior *P*. *falciparum* infection before *P*. *vivax* recurrence**										
	No	70/268.07	0.26	1			114/369.41	0.31	1		
	Yes	14/4.07	3.44	9.68	<0.001	[5.67; 16.53]	25/8.96	2.79	5.89	<0.001	[3.73; 9.30]
	**Year of recruitment**										
	2009	62/132.00	0.47	1			104/212.85	0.49	1		
	2010	22/140.14	0.16	0.30	<0.001	[0.18; 0.49]	35/165.53	0.21	0.42	0.001	[0.25; 0.71]
	**Bed net in house**										
	No	63/191.87	0.33	1			113/272.22	0.42	1		
	At least one	21/80.28	0.26	0.78	0.331	[0.47; 1.30]	26/106.16	0.24	0.63	0.017	[0.44; 0.92]
	**Prior ACT/CQ treatment before *P*. *vivax* recurrence**										
	No						115/368.66	0.31	1		
	Yes						24/9.72	2.47	5.96	<0.001	[3.87; 9.18]
	***P*. *vivax* density/μl at day 0**										
	<1,000	18/60.06	0.30	1			40/86.50	0.46	1		
	1,000–5,000	28/105.31	0.27	0.83	0.525	[0.47; 1.48]	35/138.44	0.25	0.59	0.023	[0.38; 0.93]
	>5,000	38/106.77	0.36	1.09	0.762	[0.63; 1.87]	64/153.44	0.42	0.88	0.551	[0.57; 1.35]
	**Years of education**										
	0 (children)	34/89.10	0.38	1			64/134.14	0.48	1		
	1–5	26/117.94	0.22	0.58	0.024	[0.37; 0.93]	38/151.47	0.25	0.58	0.020	[0.37; 0.92]
	6+	24/65.11	0.37	0.96	0.845	[0.62; 1.49]	37/92.78	0.40	0.86	0.468	[0.57; 1.29]

n/per year: number of cases per person-years. The following variables were systematically analyzed in both first- and all-recurrences models: village, sex, ethnic groups, age, age groups, occupation, years of education, bed net in house, economic levels, year of entry, season of entry, parasites density at day 0, prior *P*. *falciparum* infection before *P*. *vivax* recurrence, and prior ACT/CQ treatment before *P*. *vivax* recurrence. **Abbreviations**: ACT, artemisinin-based combination therapy; CGT, Conditional Gap Time; CQ, chloroquine; HR, Hazard Ratio; IR, Incidence Rate; PH, Proportional Hazard.

### Probability of remaining free of vivax recurrence

**Fig 2:**
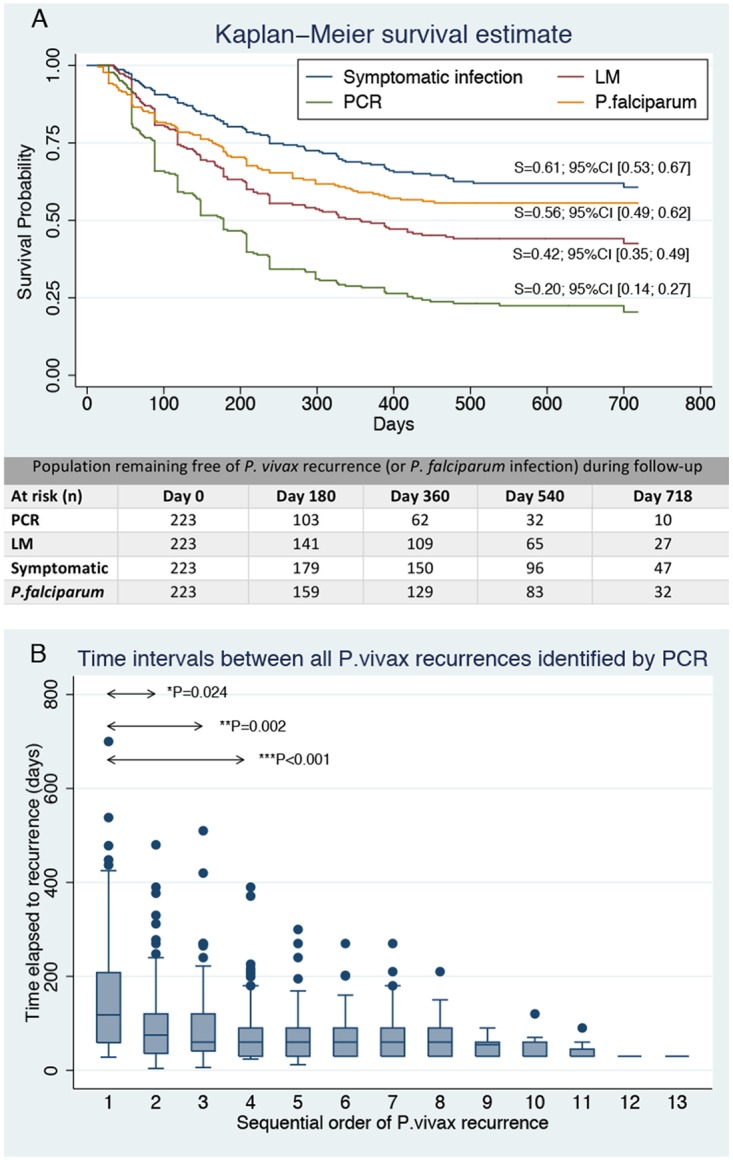
A) Overall Kaplan–Meier probability of remaining free of *P*. *vivax* recurrences (either all PCR-identified, patent, or symptomatic recurrences) and remaining free of *P*. *falciparum* infection (by PCR) by month 24 of follow-up (*n* = 223); B) time intervals between consecutive *P*. *vivax* recurrences identified by PCR (*n* = 223 patients). Wilcoxon sign rank *P*-values were computed. *Second time interval was compared to first among patients with at least two recurrences; **third time interval was compared to first among patients with at least three recurrences; ***fourth interval compared to first among patients with at least four recurrences. LM, light microscopy; PCR, polymerase chain reaction; S, survival probability.

The Kaplan–Meier probability of remaining free of recurrences by month 24 was 20.40% (95% CI [14.42; 27.13]) by PCR, 42.52% (95% CI [35.41; 49.44]) by LM, and 60.69% (95% CI [53.51; 67.11]) for symptomatic recurrences (Fig 2A). For *P*. *falciparum* recurrences, such probability was 55.61% (95% CI [48.79; 61.90]) by PCR. The median time of remaining free of PCR-detected recurrence was 174 days—i.e., half of the cohort had experienced a first recurrence by the sixth month of follow-up, while this was more than double (356 days) for patent recurrences. Most of the first events for the three types of recurrences occurred during the first year, with little changes in the cumulative risk afterwards.

The median time to first recurrence was 118 days (IQR 59–208) by PCR, 141 days (IQR 86–237) by LM, and 181 days (IQR 97–316) for symptomatic recurrence. Moreover, the median time to first recurrence tended to be shorter (88 days [IQR 58–1,784]) among patients with multiple PCR recurrences compared to those with a single PCR recurrence (163 days [IQR 58–291]; Wilcoxon rank sum *P* < 0.001 [S1 Data]). Time between PCR-detected recurrences tended to decrease with increasing number of events (Fig 2B); median times between first and second recurrence (median = 78 days; IQR 43–120) and between second and third recurrence (median = 60 days; IQR 40–120) were significantly shorter than that to first recurrence (patients with at least 2 recurrences: median = 88 days, IQR 58–174, *P* = 0.024; patients with at least 3 recurrences: median = 88 days, IQR 58–148, *P* = 0.002). From the fourth to the ninth recurrence, the median time intervals remained stable at around 60 days, and from the 10th to the 13th recurrence, at 30 days. A similar pattern was observed with patent recurrences, the median time intervals stabilizing around 76 days from the third recurrence onwards (S1 Data).

**Fig 3:**
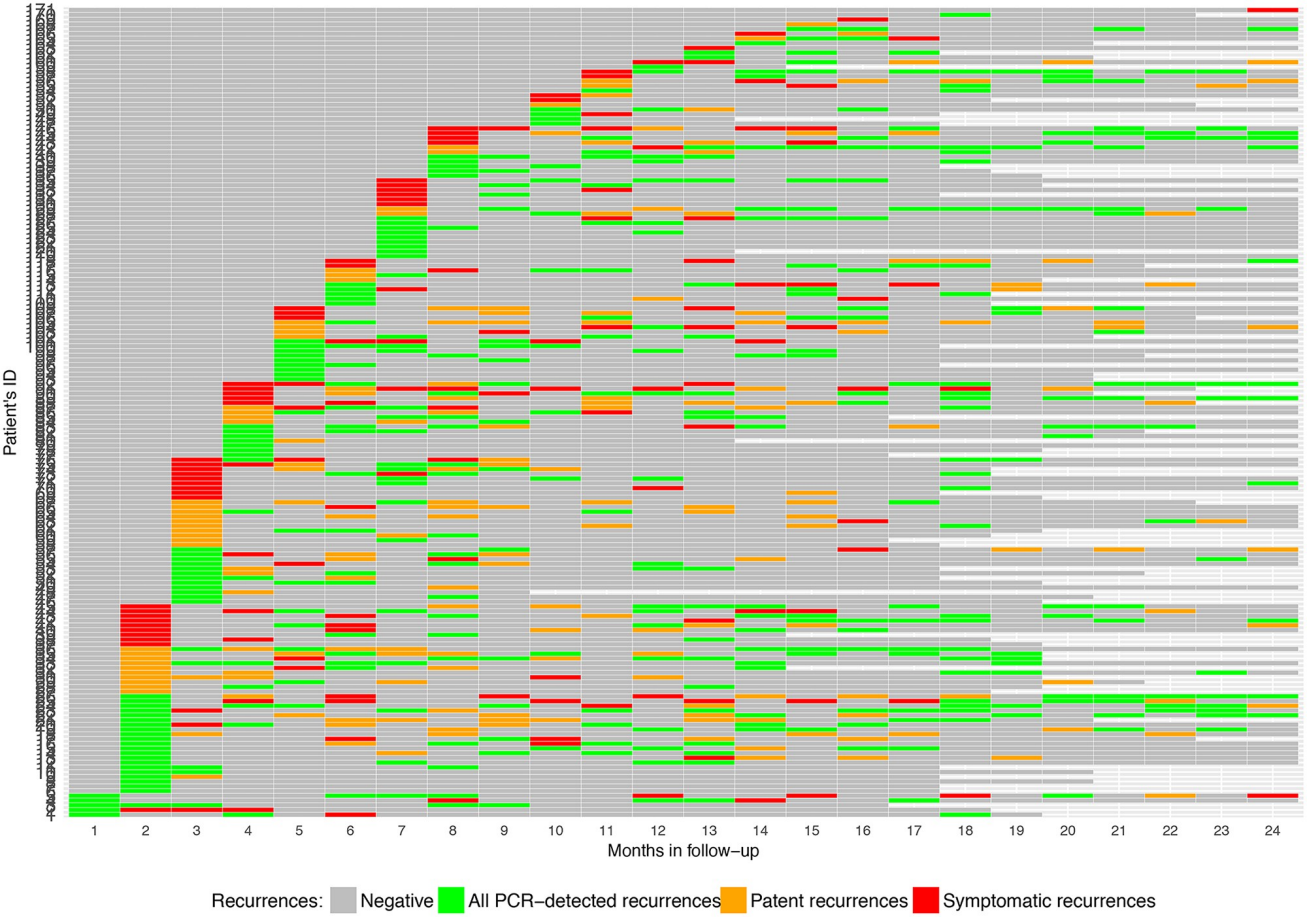
Heatmap of *P*. *vivax* recurrences by month of follow-up among the 171 cohort patients with at least one recurrence. ID, identifier; PCR, polymerase chain reaction.

Fig 3 shows a heatmap of all patients’ recurrences by month of follow-up ordered by the timing of the first recurrence. SM recurrences were frequent in almost all patients and persisted for 2–3 months (up to 7 months) before either clearing spontaneously or becoming patent or symptomatic. The frequency of symptomatic and patent recurrences decreased with increasing follow-up time, with a large majority of SM recurrences detected between month 18 and 24 of follow-up.

### Risk factors for *P*. *vivax* recurrences

Potential risk factors for a first vivax recurrence identified by the univariable analysis, regardless of the detection method, were village, ethnicity, bed nets, year of recruitment, and having a prior (1–2 months) PCR-detected *P*. *falciparum* infection (Table 3). This applied also to all *P*. *vivax* recurrences, with the additional strong effect of a prior (1–2 months) antimalarial treatment (ACT or CQ). Since village and ethnicity were highly correlated and only Village 4 (i.e., Cadong ethnicity) had a significant effect, only ethnicity was kept in the final model.

In the final Cox PH regression model, only ethnicity, prior *P*. *falciparum* infection, and year of recruitment remained significantly associated with the risk of first PCR-detected recurrence (Table 4). In addition, for the Cox PH assumption to be verified, the analysis had to be stratified by year of recruitment. The main risk factor identified for both first and all vivax recurrences was a prior *P*. *falciparum* infection, which increased by 5- to 7-fold the hazard of vivax recurrence. However, this effect was not homogenously distributed among all patients because there were significant interactions with ethnicity/Village 4 (final model for first recurrence) and prior antimalaria treatment (final model for all recurrences). Indeed, among patients recruited in 2009, the hazard of having first PCR-detected recurrence after a prior falciparum infection was 3-fold higher among Cadong (HR = 2.98; 95% CI [1.48; 5.99]; *P* = 0.002) and at least 6-fold higher among M’nong (HR = 99.19; 95% CI [6.16; 1,597.55]; *P* = 0.001) patients. Conversely, the hazard of first PCR recurrence was 2-fold higher in Cadong than in M’nong patients (HR = 2.21; 95% CI [1.33; 3.67]; *P* = 0.002), but only in those without a prior falciparum infection. In 2010, few Cadong patients were recruited, and thus the effect of ethnicity/Village 4 was no longer significant; thus, only prior falciparum infection remained significantly associated with the hazard of both first PCR-detected and patent recurrence. None of the patients recruited in 2010 had a falciparum infection before their first symptomatic recurrence.

**Table 4:** Multivariable-adjusted risk factor analysis using Cox PH model for first recurrence and Cox CGT for all recurrences.

First recurrence		2009 Adjusted			2010 Adjusted		
		HR	*P*-value	95% CI	HR	*P*-value	95% CI
**All PCR-detected recurrences**	**Effect of ethnicity according to prior *P*. *falciparum* infection before *P*. *vivax* recurrence:**				Effect of ethnicity = nonsignificant		
	No *P*. *falciparum* infection	2.21	0.002	[1.33; 3.67]	No		
	*P*. *falciparum* infection	0.07	0.059	[0.003; 1.11]			
	**Effect of prior *P*. *falciparum* infection before *P*. *vivax* recurrence according to ethnic groups:**				**Effect of prior *P*. *falciparum* before *P*. *vivax***		
	M’nong	99.19	0.001	[6.16; 1,597.55]	1		
	Cadong	2.98	0.002	[1.48; 5.99]	5.33	0.012	[1.45; 19.62]
**Patent**	**Prior *P*. *falciparum* infection before *P*. *vivax* recurrence**						
	No	1			1		
	Yes	6.51	<0.001	[3.66; 11.56]	5.92	0.018	[1.36; 25.75]
**Symptomatic**	**Prior *P*. *falciparum* infection before *P*. *vivax* recurrence**						
	No	1					
	Yes	7.83	<0.001	[4.25; 14.44]			
**All recurrences**		**2009**			**2010**		
**All PCR-detected recurrences**	**Effect of malaria treatment according to prior *P*. *falciparum* infection before *P*. *vivax* recurrence:**						
	No *P*. *falciparum* infection	6.35	<0.001	[4.32; 9.33]	8.94	<0.001	[5.07; 15.77]
	*P*. *falciparum* infection	0.72	0.118	[0.47; 1.09]	1.53	0.482	[0.47; 5.02]
	**Effect of prior *P*. *falciparum* infection before *P*. *vivax* recurrence according to malaria treatment:**						
	No malaria treatment	5.89	<0.001	[4.34; 7.98]	5.06	<0.001	[2.57; 9.96]
	Malaria treatment	0.66	0.09	[0.41; 1.07]	0.87	0.800	[0.29; 2.63]
**Patent**	**Effect of malaria treatment according to prior *P*. *falciparum* infection before *P*. *vivax* recurrence:**						
	No *P*. *falciparum* infection	21.40	<0.001	[10.61; 43.18]			
	*P*. *falciparum* infection	0.77	0.365	[0.44; 1.35]			
	**Effect of prior *P*. *falciparum* infection before *P*. *vivax* recurrence according to malaria treatment:**						
	No malaria treatment	6.34	<0.001	[4.21; 9.54]			
	Malaria treatment	0.23	<0.001	[0.10; 0.50]			
	**Prior *P*. *falciparum* infection before *P*. *vivax* recurrence**						
	No				1		
	Yes				1.80	0.07	[0.96; 3.37]
	**Prior ACT/CQ treatment before *P*. *vivax* recurrence**						
	No				1		
	Yes				13.83	<0.001	[3.85; 49.72]
**Symptomatic**	**Effect of malaria treatment according to prior *P*. *falciparum* infection before *P*. *vivax* recurrence:**						
	No *P*. *falciparum* infection	6.22	<0.001	[2.80; 13.82]			
	*P*. *falciparum* infection	1.07	0.88	[0.45; 2.54]			
	**Effect of prior *P*. *falciparum* infection before *P*. *vivax* recurrence according to malaria treatment:**						
	No malaria treatment	5.65	<0.001	[2.81; 11.35]			
	Malaria treatment	0.97	0.95	[0.38; 2.46]			
	**Prior ACT/CQ treatment before *P*. *vivax* recurrence**						
	No				1		
	Yes				6.05	0.001	[2.17; 16.86]

Among patients recruited in 2009, a significant interaction term (HR = 0.03; *P* = 0.016) for the hazard of first PCR-detected recurrence was found between ethnicity and prior *P*. *falciparum* infection. Similarly, significant interaction terms were found for the hazard of all PCR-detected recurrences (HR = 0.11, *P* < 0.001); patent recurrences (HR = 0.035, *P* < 0.001) and symptomatic recurrences (HR = 0.17, *P* = 0.03). For patients recruited in 2010, a significant interaction term (HR = 0.17; *P* = 0.008) for the hazard of all PCR-detected recurrences was found between prior *P*. *falciparum* infection and prior antimalaria treatment. **Abbreviations**: ACT, artemisinin-based combination therapy; CGT, Conditional Gap Time; CQ, chloroquine; HR, Hazard Ratio; PCR, polymerase chain reaction; PH, Proportional Hazard.

Similarly, the final Cox CGT models for all recurrences among patients recruited in 2009 and 2010 showed that the hazard of PCR-detected vivax recurrences after a prior falciparum infection (versus no *P*. *falciparum*) was 5- to 6-fold higher, but only in patients without previous antimalaria treatment (i.e., SM falciparum infections). Conversely, the effect of prior antimalarial treatment (versus no treatment) was very strong in both recruitment years (respectively, HR = 6.35 and HR = 8.94; *P* < 0.001), but only in individuals without a prior falciparum infection, i.e., patent recurrences treated with CQ. For patent and symptomatic recurrences, a similar pattern was observed for patients recruited in 2009.

**Fig 4:**
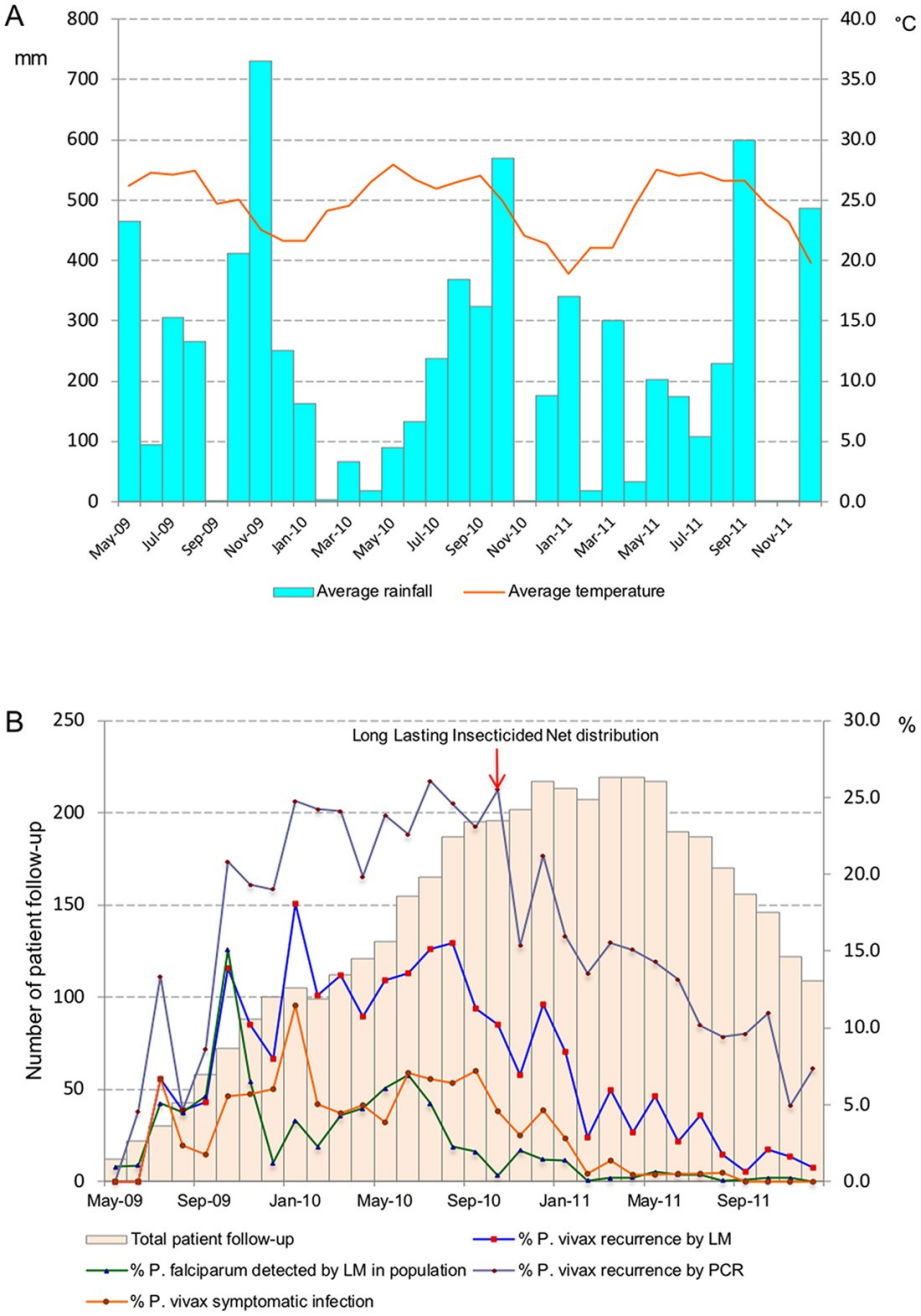
A) Monthly climatic data in the study area; B) monthly evolution of the cohort size with proportion of patients identified with *P*. *vivax* recurrence (all PCR-identified, all patent, and all symptomatic) and the monthly incidence risk of *P*. *falciparum* infections detected by ACD and PCD (LM) in study population. ACD, active case detection; LM, light microscopy; PCD, passive case detection.

The hazard of patent recurrences was significantly higher (HR = 6.34; *P* < 0.001) in patients with a prior untreated falciparum infection, while the opposite effect was observed in patients who were treated (HR = 0.23; *P* < 0.001). For patients recruited in 2010, only prior antimalarial treatment remained significantly associated with the risk of patent (HR = 13.83; *P* < 0.001) and symptomatic recurrences (HR = 6.05; *P* < 0.001). Fig 4A shows the study timeline with corresponding climatic data; Fig 4B shows the evolution of the cohort size by calendar month together with the proportion of study patients with vivax recurrences (PCR-detected, patent, and symptomatic) as well as the contemporary monthly incidence risk of *P*. *falciparum* infections (Fig 4B) as detected by PCD and ACD (LM) in the four study villages. The first mass distribution of long-lasting insecticidal nets (LLINs) in the study area occurred in September 2010 and was followed by a steady decrease in both the risk of *P*. *falciparum* infections in the study area and of *P*. *vivax* recurrences among cohort patients (Fig 4B). The monthly incidence risk of *P*. *falciparum* decreased to almost zero from February 2011 until the end of follow-up; similarly, the risk of all PCR-detected and patent vivax recurrences steadily decreased to 7% and 1%, respectively, by December 2011.

## Discussion

Post-treatment vivax recurrences were extremely frequent, especially SM infections, which tended to occur earlier than patent or symptomatic infections. Prior falciparum infection was the main risk factor for all types of vivax recurrence (first and all recurrences; PCR-detected, patent, and symptomatic recurrences) together with prior CQ treatment for the risk of repeated recurrences. The drop-out rate was low given the length of follow-up, and most withdrawals occurred during the 10-day PQ treatment.

Although collected about ten years ago, our results remain relevant for Vietnam where the current challenges for malaria elimination include i) the increasing relative frequency of *P*. *vivax* malaria, from 25% of all cases in 2009 to 51% in 2015 [9]; and ii) the spread of artemisinin resistance [29]. In addition, the downward trend of annual malaria incidence has changed as the total number of confirmed cases started to increase in 2017, with a 16% increase observed in 2018 (4,813 cases) compared to 2016 (4,161 cases) [30,31], mostly due to *P*. *falciparum*; cases were observed mainly in a few provinces in Central Vietnam where artemisinin resistance has been reported [32].

Despite PQ directly observed treatment, 8% of patients (most withdrawals) withdrew their consent during the 10-day treatment, emphasizing the difficulty of achieving good compliance for PQ radical treatment [18,33]. In our cohort, PQ was only used at the time of recruitment and at the end of each patient’s follow-up. This is probably similar to the true situation in remote areas in Vietnam, including our study area, where the health staff is reluctant to administer PQ in the absence of diagnostic tests for G6PD deficiency [16]. Tafenoquine, a new 8-aminoquinoline with prolonged half-life recently approved by the US Food and Drug Administration [3], will not change this situation because it will be administered only after performing a quantitative G6PD rapid test [34], although the single-dose scheme will improve compliance.

Incidence of PCR-detected vivax recurrence was >2-fold higher than that of patent recurrence and 5-fold higher than symptomatic recurrence, suggesting a much larger infection reservoir than estimated by standard diagnostic tools (LM/RDT) and current surveillance strategies (PCD and Re-ACD). Although some of the PCR-detected infections at different time points may be the same infection, possibly overestimating the incidence of recurrent infections, their persistence for long periods of time (up to 7 months) contributes to maintaining transmission. Our results corroborate the findings of a recent cohort study in Central Vietnam in which the median duration of SM *P*. *vivax* infections was 6 months (IQR 3–9), with parasite densities oscillating from ultralow to high density between monthly samples [35]. Since SM vivax infections with gametocytes can successfully infect mosquitoes [36,37], all PCR-detected vivax infections should be considered as potentially infectious, regardless of parasite density or presence of gametocytes at time of sampling, and effectively treated in order to interrupt transmission.

Although the annual malaria incidence reported by PCD has further decreased since 2011, with no falciparum malaria cases since 2016 (S2 Table), the current proportion of SM vivax infections may be similar to that observed in our study. Indeed, during the last 6 months of our cohort study, in 2011, when malaria transmission had decreased substantially, PCR-detected vivax recurrences were significantly more frequent than patent or symptomatic infections. In a recent systematic review [38], SM vivax infections probably represent about 67% of all PCR-detected vivax infections and are negatively correlated, as for *P*. *falciparum* [22], with the prevalence of patent vivax infections. This suggests that, despite the observed reduction of patent vivax and falciparum cases since 2011, the proportion of SM vivax infections may not have changed; this should be further confirmed by large-scale prevalence surveys using both PCR and LM.

*P*. *falciparum* infections were identified as the main risk factor for first and repeated vivax recurrences. The triggering effect of symptomatic *P*. *falciparum* infections on *P*. *vivax* relapses has been known since the 1920s [39] and has been reported in other cohort studies in Asia [40–42]. Interestingly reported and for the first time, to our knowledge, in our study, falciparum infections were a strong risk factor for repeated vivax recurrences, but only in patients untreated (with either CQ or DHA-PQ) at the previous visit, while if treated, falciparum infection tended to be protective. This suggests that chronic SM falciparum infections can trigger vivax recurrences, while antimalarial treatment, by clearing falciparum infections, prevents vivax recurrence. Though it remains unclear whether *P*. *falciparum* triggered vivax relapses or suppressed vivax parasites during coinfections, our results suggest that chronic SM falciparum infections could trigger vivax recurrences, hence contributing to the human reservoir of *P*. *vivax* infections. This, together with previous evidence [40–42], calls for new and integrated treatment strategies tackling both species simultaneously, e.g., treating all malaria infections, regardless of the species, with one ACT and antirelapse drug (PQ or tafenoquine) [43]. The increased risk of vivax recurrence related to previous antimalarial treatment in patients without prior falciparum infection suggests CQ vivax resistance as confirmed earlier in the same cohort [44].

It is not possible to distinguish between new infections and relapses within our cohort. Nevertheless, assuming a similar incidence of *P*. *falciparum* and *P*. *vivax* new infections, the difference between the PCR-detected vivax and falciparum infections’ Kaplan–Meier curves should provide the relative contribution of vivax relapses, i.e., around 50%. This compares well with the results of a trial comparing artesunate alone or combined with PQ in Papua New Guinea, where *P*. *falciparum* and *P*. *vivax* sporozoite rates in the local vector population were comparable [45].

The total PQ dose administered (5.0 mg/kg) was lower than that recommended by WHO, i.e., 7.0 mg/kg [46]. A recent systematic review reported that a total PQ dose ≥5.0 mg/kg had the lowest risk of recurrence (median = 0%; range 0%–15%) than the very low (≤2.5 mg/kg) and low total dose (>2.5 and <5.0 mg/kg) [5]. This confirmed a previous review concluding that 0.5 mg/kg/day for 14 days was the recommended dose in nonpregnant subjects without G6PD deficiency [47]. Although it is impossible to know whether a 2.0 mg/kg difference in PQ dosage would have affected the risk of first recurrence, the synergistic effect of combining CQ with PQ could have been hampered by *P*. *vivax* CQ resistance, which was observed in our cohort [44]. This is also suggested by the strong deleterious effect of prior CQ treatment on all types of vivax recurrence in 2009 and for the PCR-detected recurrences in 2010. CQ resistance in *P*. *vivax* should be carefully monitored in Vietnam, and treatment for vivax patients changed to an ACT in case of substantial resistance [48,49].

A major limitation of this study is the absence of a comparator arm in which all vivax recurrences would have been systematically treated with PQ; this would have enabled estimating the relative contribution of vivax relapses to recurrent infections [45]. However, such a design was not chosen given the low incidence rates of symptomatic vivax infections (main inclusion criteria), which would have required an even longer recruitment period that could not be supported by the available budget.

As mentioned earlier, the age of the data represents another limitation because malaria continued to decrease steadily after the end of the study, with no falciparum cases reported in 2016 and 2017 (S2 Table). However, as mentioned earlier, *P*. *falciparum* is on the rise in Central Vietnam, and the province of Quang Nam also reported an increase in *P*. *falciparum* cases in 2018 (+4% compared to 2017). Therefore, even though the data presented in this paper are not directly comparable to the current situation in the study area, the challenges they underline, i.e., the elimination of *P*. *vivax* and of artemisinin-resistant *P*. *falciparum* in Central Vietnam, remain more than ever relevant.

Because hemoglobin (Hb) levels were not monitored during the follow-up, it was not possible to determine the risk and severity of anemia related to vivax recurrences, especially for the prolonged SM vivax infections, which was another major limitation. Finally, in our statistical analysis, the different sampling schemes underlying scheduled and unscheduled visits were not considered. Nevertheless, the related bias is probably minor because of the intense monthly sampling and the moderate number of unscheduled visits (4.533 scheduled visits versus 71 unscheduled visits). Further research should focus on developing appropriate methodology taking into account the outcome-dependent sampling underlying unscheduled events, especially in case of larger time gaps between scheduled visits [50].

In conclusion, this large cohort study identified a high burden of post-treatment vivax infections, mainly asymptomatic and SM, despite a high-dose PQ regimen (total 5 mg/kg) administered under direct observation. The main risk factors for vivax recurrence were prior *P*. *falciparum* infections—particularly the SM ones, which were untreated because they were not detected immediately after sampling and persisted for several months—and prior CQ monotherapy, indicating vivax drug resistance. When considering Vietnam reverted to the standard PQ dose of 0.25 mg/kg/day for 14 days, which is often not prescribed or supervised, the current vivax parasite reservoir, despite the decreasing number of reported cases, may be larger than estimated by standard diagnostic tools. This calls for improved treatment (e.g., single-dose tafenoquine with G6PD testing) and surveillance strategies (including the detection of SM infections) tackling simultaneously *P*. *falciparum* and *P*. *vivax* infections if malaria elimination is to be achieved by 2030 as endorsed by the Vietnamese government.

## Supporting information

S1 Table Baseline characteristics of the 260 patients enrolled in the cohort split by the year of recruitment. (XLSX)

S2 Table Incidence of malaria in four study villages from 2012 to 2018. (XLSX)

S1 Data Data of Tables 2, 3 and 4 and Figs 2 and 3. (XLSX)

S1 Fig Data of Fig 1. (XLSX)

S2 Fig Data of Fig 4. (XLSX)

S1 Text Protocol: *P*. *vivax* epidemiology and control in Vietnam. (DOC)

S2 Text STROBE statement. STROBE Statement is a checklist of 22 items that we consider essential for good reporting of cohort studies. These items relate to the article′s title and abstract (item 1), the introduction (items 2 and 3), methods (items 4–12), results (items 13–17) and discussion sections (items 18–21), and other information (item 22 on funding). STROBE, Strengthening The Reporting of OBservational Studies in Epidemiology.
(DOC)

## Abbreviations

ACT: artemisinin-based combination therapy
CGT: Conditional Gap Time
CHC: Commune Health Center
CQ: chloroquine
DHA-PQ: dihydroartemisinin-piperaquine
FPBS: filter paper blood sample
G6PDd: Glucose-6-Phosphate-dehydronage deficient
Hb: Hemoglobin
HHW: Hamlet Health Worker
HR: Hazard Ratio
ITMA: Institute of Tropical Medicine, Antwerp
LCF: late clinical failure
LLIN: long-lasting insecticidal net
LM: light microscopy
LPF: late parasitological failure
NIMPE: National Institute of Malariology, Parasitology, and Entomology
NMCEP: National Malaria Control and Elimination Program
PCD: passive case detection
PCR: polymerase chain reaction
PH: Proportional Hazard
PQ: primaquine
RDT: rapid diagnostic test
Re-ACD: re-active case detection
SM: submicroscopic
SnM: Seminested Multiplex
TB: tuberculosis
WBC: white blood cell
WHO: World Health Organization
