# Supplementary material for: Plasmodium vivax morbidity after radical cure: A cohort study in Central Vietnam
Source: PLoS Med. 2019 May 17;16(5):e1002784. doi: 10.1371/journal.pmed.1002784 (PMC6524795; doi:10.1371/journal.pmed.1002784)
Supplement: S1 Text — (DOC) [file pmed.1002784.s006.doc]

***Plasmodium vivax* genotyping and modelling:**

**a new tool for malaria control**

**in Central Vietnam**

***Protocol version 3.1***

***25.02.2010***

**FULL TITLE**

*Plasmodium vivax* genotyping and modelling: a new tool for malaria control in Central Vietnam

**SHORT TITLE:**

*P.vivax* epidemiology & control in Vietnam

**PRINCIPAL INVESTIGATORS:**

**Le Xuan Hung, MD, PHD**

National Institute of Malariology, Parasitology & Entomology (NIMPE)

Luong The Vinh street 245

BC10200 - Tu Liem district

Hanoi, Vietnam

Tel/Fax: +84 4 5532818/+8448543015

E-mail: [lxhung1952@yahoo.com](mailto:lxhung@yahoo.com)

**Annette Erhart, MD, Msc, PhD**

Prince Leopold Institute of Tropical Medicine

Nationalestraat 155

B-2000 Antwerp-**Belgium**

Tel/Fax: 0032 3 247 6308/59

E-mail: [aerhart@itg.be](mailto:aerhart@itg.be)

**CO-INVESTIGATORs:**

***- Field coordinator:***

**Pham Vinh Thanh, MD, Msc**

NIMPE

Luong The Vinh street 245

BC10200 - Tu Liem district

Hanoi, Vietnam

Tel/Fax: +84 4

E-mail: [phamvinht@gmail.com](mailto:phamvinht@gmail.com)

***- Laboratory coordinator, NIMPE***

**Dr Nguyen Van Hong, MD**

NIMPE

Luong The Vinh street 245

BC10200 - Tu Liem district

Hanoi, Vietnam

Tel: +84 4

Email: [nvhong1982@yahoo.com](mailto:nvhong1982@yahoo.com)

***- Project manager, NIMPE:***

**Ngo Duc Thang, MD, Msc**

NIMPE

Luong The Vinh street 245

BC10200 - Tu Liem district

Hanoi, Vietnam

Tel/Fax: +84 4

E-mail: [thangnimpevn@yahoo.com](mailto:thangnimpevn@yahoo.com)

***- Laboratory coordinator, ITM***

**Peter Van den Eede, Msc**

Prince Leopold Institute of Tropical Medicine

Nationalestraat 155

B-2000 Antwerp-**Belgium**

Tel/Fax: 0032 3 247 6488/59

***- Co PI:***

**Umberto D’Alessandro, MD, Msc, PhD**

Prince Leopold Institute of Tropical Medicine

Nationalestraat 155

B-2000 Antwerp-**Belgium**

Tel/Fax: 0032 3 247 6354/59

E-mail: [udalessadro@itg.be](mailto:udalessadro@itg.be)

**INSTITUTIONS:**

1. **National Institute of Malariology, Parasitology & Entomology** **(NIMPE)**

Luong The Vinh street 245

BC10200 - Tu Liem district

Hanoi, Vietnam

1. **Prince Leopold Institute of Tropical Medicine (ITM)**

Nationalestraat 155

2000 Antwerp

BELGIUM

**DATES:**

**start:** April 2009

**end:** September 2011

**CORRESPONDENCE TO:**

**Annette Erhart,** ITM

Tel/Fax: + 32 3 247 63 08/(59)

E-mail: aerhart@itg.be

**ACRONYM: VIVA_VIETNAM**

**Approval Signature Date**

Prof./Dr. …………..……............_______________________________ ____/____/___

Investigator

Prof./Dr. …………..……............ _______________________________ ____/____/___

Investigator

Prof./Dr. …………..……............ _______________________________ ____/____/___

Investigator

Prof./Dr. …………..……........... _______________________________ ____/____/___

Investigator

Prof./Dr. …………..…….......... _______________________________ ____/____/___

Investigator

Prof./Dr. …………..…….......... _______________________________ ____/____/___

Investigator

Prof./Dr. …………..………….. _______________________________ ____/____/___

Investigator

**SUMMARY**

*Plasmodium vivax* is the most widely spread of the four malaria species and the main cause of malaria outside Sub-Sahara Africa. In Central Vietnam, nearly 50% of malaria infections are due to *P.vivax*, causing a heavy morbidity burden for the local population due to the activation (relapse) of dormant liver forms (hypnozoites) weeks or months after the primary infection. The national guideline for the radical cure of *P.vivax* infection was changed in 2007, with an increase of the daily dose of primaquine from 0.25mg/kg to 0.50mg/kg for 10 days (the 3-day concomitant chloroquine regimen (total 25mg/kg) remaining unchanged). The efficacy of this new regimen in preventing short as well as long term relapses has not been evaluated yet. Moreover, the epidemiology and the transmission dynamics of *P.vivax* remains poorly documented due to the lack of reliable tools to distinguish between new infections, recrudescence and relapses. Consequently, as control strategies cannot be adequately adapted to the local epidemiology, *P.vivax* morbidity continues to contribute to the vulnerability of the local populations, mainly ethnic minorities, with poor health and socio economic status. The main objective of this study is to establish a new tool that will assist the National Malaria Control Program to improve control strategies for *P.vivax* in Vietnam. This tool will be based on molecular (microsatellites) and serological (CSP ELISA) techniques and on mathematical modelling, to distinguish between a new infection, and a relapse/recrudescence of *P.vivax*. A cohort of 250 *P.vivax* infected patients will be treated and followed up for 2 years in order to estimate the efficacy of the current treatment guideline in preventing recrudescence and relapses, and determine the incidence of new infections. The development of such a tool is extremely important for the control of *P.vivax* in Vietnam since it will help to understand the epidemiology of *P.vivax* and to test targeted interventions, including new drugs. Eventually, the National Malaria Control Program will be able to adapt its control strategies and monitor the efficacy of the treatment guidelines. This study will be conducted concomitantly to a similar one in the Peruvian Amazon (“sister project”), in order to compare the results and to validate the new tool. In addition, this project will contribute to strengthen the institutional collaboration between NIMPE, Hanoi and ITM, Antwerp with the training of 2 Vietnamese junior scientists (1 Msc and 1 PhD), and to develop a new South-South collaboration between the NIMPE, Hanoi and ITM-AvH, Lima, through simultaneous training of 2 Peruvian students. Exchange of information, experience and expertise on *P.vivax* between the 2 southern institutions is likely to boost new perspectives in R&D in 2 endemic regions facing similar challenges for malaria control.

**TABLE OF CONTENTS**

[1. BACKGROUND 7](#__RefHeading___Toc229122018)

[2. OBJECTIVES 8](#__RefHeading___Toc229122019)

[2.1 Main objective 8](#__RefHeading___Toc229122020)

[2.2 Specific objectives 8](#__RefHeading___Toc229122021)

[3. MATERIALS & METHODS 8](#__RefHeading___Toc229122022)

[3.1 Study site and population 8](#__RefHeading___Toc229122023)

[3.2 Study design 9](#__RefHeading___Toc229122024)

[3.2.1 Cohort recruitment 10](#__RefHeading___Toc229122025)

[3.2.2 Loss to follow-up 10](#__RefHeading___Toc229122026)

[3.2.3 Patient discontinuation or protocol violation 11](#__RefHeading___Toc229122027)

[3.2.4 Rescue treatment 12](#__RefHeading___Toc229122028)

[3.2.5 Study endpoints 12](#__RefHeading___Toc229122029)

[3.2.6 Sample size calculation 13](#__RefHeading___Toc229122030)

[3.3 Data collection 14](#__RefHeading___Toc229122031)

[3.3.1 Screening & survey 14](#__RefHeading___Toc229122032)

[3.3.2 Passive case detection of *P.vivax* patients 15](#__RefHeading___Toc229122033)

[3.3.3 *P.vivax* patients’ treatment & cohort follow-up 16](#__RefHeading___Toc229122034)

[3.4 Blood samples processing 22](#__RefHeading___Toc229122035)

[3.5 Data entry & Analysis 24](#__RefHeading___Toc229122036)

[4. ETHICAL ISSUES 24](#__RefHeading___Toc229122037)

[5. REFERENCES 25](#__RefHeading___Toc229122038)

# BACKGROUND

According to the national control program (NMCP), half of the malaria morbidity and about 90% of its mortality occurs in the 16 forested provinces situated in Central Vietnam (1, 2). In these areas, *Plasmodium vivax* represents about half of all malaria cases (3, 4), and the local populations bear a heavy morbidity burden due to the activation (relapses) of dormant liver forms (hypnozoites) weeks or months after the primary infection. Hypnozoites can be treated with a 2-week course of primaquine as recommended by WHO (5). However, treatment adherence is usually low and self-treatment with inappropriate drugs or inadequate dosage is common as symptoms are often mild. Therefore, Vietnam has recently (2007) doubled the daily dose of Primaquine (from 0.25 to 0.5mg/kg) in its 10-day protocol (6). However, the efficacy of this new regimen in preventing short- and long-term relapses has not yet been formally evaluated. Moreover, the epidemiology of *P.vivax* is poorly documented because the currently available techniques cannot discriminate between relapses/recrudescences and new infections. Therefore, the incidence of new infections and related risk factors cannot be estimated, the current treatment efficacy and the impact of control activities cannot be accurately evaluated and new interventions cannot be tested. This results in a *statu quo* as control strategies cannot be adapted to the local epidemiology, and consequently maintains the vulnerability of local populations, mainly ethnic minorities with poor health and socio-economic status. We propose to set up a new tool that will assist the NMCP in understanding the transmission dynamics of *P.vivax*, in monitoring the treatment efficacy, and in designing and evaluating targeted strategies to control *P.vivax* malaria. This method will combine molecular, serological and epidemiological markers that will be incorporated in a mathematical model to estimate the probability for a given vivax episode to be a new infection. Similar models have already been developed for *P. falciparum* (7) and will be adapted for *P. vivax*. Currently, the best molecular markers for *P.vivax* genotyping are micro-satellites (MS), which are highly polymorphic tandem repeats. However, genotyping alone is not sufficient since a true relapse could have a different genetic pattern from the primary infection (8), due to different parasite clones present in the liver. Thus, recent exposure to an infective mosquito bite needs also to be checked by measuring antibody titres against *P. vivax* circumsporozoite protein (CSP), short-lived (1 month) and specific against sporozoites. This can be done with an existing CSP Enzyme Linked Immunosorbent Assay (ELISA) (9-10). MS markers and CSP-ELISA are currently under development at ITM, Antwerp.

# OBJECTIVES

## Main objective

To improve malaria control strategies in Central Vietnam by characterizing *P.vivax* epidemiology and identify potential targets for intervention.

## Specific objectives

1. To establish in a defined cohort of *P.vivax* patients, the short and long term efficacy of the new national treatment guideline in preventing recrudescences and relapses;
2. To characterize *P.vivax* transmission dynamics and indentify vulnerable groups among the local population (in a representative sample of the study population);
3. To set up a new tool, based on genotyping, serology and modelling able to accurately distinguish between *P.vivax* new infections and relapses.

# MATERIALS & METHODS

## Study site and population

The study will be carried out in the 2 neighbouring communes of Tra Leng and Tra Don situated in Nam Tra My district, Quang Nam Province, Central Vietnam, where several malaria outbreaks have occurred in the past, and currently with most *P.vivax* cases in the province (11). The latter come mostly from Villages 1, 2 and 3 in Tra leng and from Village 3 in Tra Don commune. The commune health centre (CHC) of Tra leng is situated in Village 1, which is contiguous to Village 3, while Village 2 is situated at 2h walking distance through the forest on the top of a mountain. Village 4 is located even further (4h walking distance) and will be included in the study area in a second phase (March 2010) in order to increase the recruitment rate. The area is still densely forested and mountainous, a perfect site for *Anopheles dirus*, though the main vector species identified in entomological collections by the Provincial Malaria Station (PMS) is *An.minimus A,* together with other species such as *An.vagus, An.aconitus,* or *An.philippinensis* (12).

The climate is sub-humid with an average annual rainfall of about 1,200-2,000mm and two seasons: the dry season from December to April, and rainy season from May to November. Malaria transmission is perennial with 2 peaks: the first at the beginning of the rain (March-May), and the second between August and October. Annual reports from the national program show that about 40 to 45% of the malaria cases are due to *P.vivax* and all age groups are infected. The 2 communes totalled up to 400 and 300 cases, respectively in 2005 and 2006, with a total annual incidence of about 150 *P.vivax* cases/1000 populations. In 2007, the incidence dropped to 153 cases, and in 2008 only 106 cases were detected. Despite the decreasing incidence, more cases can probably be found by systematic screening.

The population of the 4 initial study villages comprises about 2,100 inhabitants (370 households), 85% belonging to the M’nong ethnic minority and 15% to the Cadong, clustered into small hamlets of 15-20 houses (February 2009 Census). The census of Village 4 will be carried out in March 2010. People are usually very poor, living in bamboo or wooden houses on the slope of the mountains. Most people practice subsistence agriculture, cultivating maize, manioc and rice in their forest fields and for the richer ones planting cinnamon trees. Most people rear goats and pigs and for those who can afford it cattle and buffaloes. ITN use is not very popular among the M’nong as they fear burning them because they usually sleep near the fire. Each commune has a CHC staffed by a few nurses, one microscopist (3-month training) and a midwife. They supervise the hamlet health workers (usually one per hamlet) who are involved in health education and prevention activities. They are also trained to take blood slides from febrile patients and bring them to the CHC for reading. Whenever a slide is found positive for malaria parasites, the patient is referred to the CHC for treatment. Quality control of the microscopic examination is done at district and at provincial level.

## Study design

This is a prospective longitudinal study of a cohort of *P.vivax* infected patients initially treated with the radical cure regimen following national guidelines and followed-up monthly for 2 years.

### Cohort recruitment

Vivax infected patients will be recruited during the initial screening/survey and/or during one year of the passive case detection (PCD) continued in each study village during the whole cohort follow-up.

Initially, the recruitment period was foreseen from April 2009 to April 2010. Since only 117 *P.vivax* patients could be recruited by February 2010, it was decided to extend the recruitment until December 2010 in order to reach the target number of 250 patients included.

*Inclusion criteria*

- Age ≥ 3years
- *P.vivax* mono-infection (asexual stages);
- Resident in the study villages;
- Written informed consent (parent/guardian for minors);

*Exclusion criteria*

- General danger signs with severe or complicated malaria according to WHO definitions (Annex B to this protocol);
- Pregnancy (rapid test positive or pregnancy clinically confirmed) or breastfeeding;
- Known G6PD deficiency (or history of “black urine” after previous primaquine treatment), or any history of side effects to the study drug;
- Documented radical cure with Primaquine (10 days at 0.5mg/kg) within one month prior to inclusion;
- Underlying chronic diseases (TB, lupus, diabetes, rheumatoid arthritis, etc);
- Concomitant infectious disease requiring antibiotic treatment;
- Severe malnutrition;
- Patients with neuro-psychiatric disorders or previous history (epilepsy, dementia, ..)

### Loss to follow-up

Loss to follow up occurs when, despite all reasonable effort, an enrolled patient that does not attend the scheduled visits cannot be found. No treatment outcome will be assigned to these patients during the 28-day follow-up. These patients will be classified as lost to follow-up. Patients who are lost to follow-up, but who subsequently come back to the study site before the end of the 28-day follow-up (or the end of the 2-year follow-up), will not be turned away and will also be encouraged as much as possible to return into the follow-up. The reason and location (geographical) of the absence must be written in the follow-up forms Annex 7 or Annex8, accordingly). These patients will be classified as “temporarily lost”.

Every effort must be taken to trace the patients for their follow-up visits with the help of the HHW (and community members) during the entire follow-up period.

### Patient discontinuation or protocol violation

Cohort patients meeting any of the following criteria will be classified as **withdrawn**

- **Withdrawal of consent:**

Any patient may withdraw consent at any time, without prejudice for further follow-up or treatment in the study site.

- **Failure to complete the treatment due to either:**
- Failure to attend the scheduled visits during the 10-day treatment;
- Serious adverse events necessitating termination of the treatment before the full course (10 days) of the treatment is completed;

A patient who vomits twice the study medications will be withdrawn from the study and given the rescue treatment.

A patient can be discontinued from the study if the investigator decides that a patient should be withdrawn for safety reasons due to serious adverse events (signs of hemolytic anemia (yellow conjonctives/dark urine) or Grade 3 to 4 of the WHO classification for adverse events). In this case, information concerning the adverse event and symptomatic treatment given must be recorded on the patient follow-up form (Annex 7).

- **Voluntary protocol violation (28-day follow-up)**

Self- or third party administration of antimalarial (or antibiotics with antimalarial activity such as tetracycline, amoxicilline).

- **Involuntary protocol violation (during the 28-day follow-up)**
- Occurrence of concomitant disease that would interfere with a clear classification of the treatment outcome;
- Detection of another malaria species during the follow-up;
- Erroneous inclusion of a patient who do not meet the inclusion criteria;
- Misclassification of a patient due to a laboratory error (parasitaemia) leading to the administration of the rescue treatment;

**Patients who are withdrawn will be followed up until day 28 and continue the 2-year monthly follow-up.** However, no treatment outcome will be assigned to these patients during the 28-day follow-up. The reason for discontinuation or protocol violation will always be recorded on the follow-up forms (Annex 7 or Annex 8, accordingly).

### Rescue treatment

- With any sign of severe malaria, the patient should be administered parenteral antimalaria treatment with either quinine or artesunate (IV) following national guidelines;
- If a patient meets one of the criteria for therapeutic failure (ETF, LTF), he/she will receive Arterakin for 3 days following the national guideline. However, this patient will remain in the 2-year monthly follow-up, and thus should be re-assessed clinically and biologically at Day 28 (following D28 procedures mentioned in Annex7), and then enter the monthly follow-up.
- If the patient is re-infected with *P.falciparum* (mono- or mixed infection) he/she will receive the rescue treatment with Arterakin for 3 days following the national guideline.

### Study endpoints

- Primary Endpoint

There will be two primary end points for determining the efficacy of the *P. vivax* national treatment guidelines:

- Treatment Failure (TF) at day 28 (see below);
- Incidence of *P. vivax* (recurrent) infection after day 28 during the 2-year follow up;

The TF is defined as the sum of early* and late** treatment failures.

- * Early Treatment Failure (ETF) (one of the following)

(i) Development of danger signs on Day 0, Day 1, Day 2 or Day 3, in the presence of parasitaemia;

(ii) Parasite density on Day 2 > Day 0 count, irrespective of axillary temperature,

(iii) Presence of parasitaemia on Day 3 with fever (axillary temperature ≥ 37.5°C),

1. Parasitaemia on Day 3 ≥ 25 % of count on Day 0.

- ** Late treatment failure (LTF)
- LTF is divided in late clinical and late parasitological failure.

Late Clinical Failure (LCF):

(i) Development of danger signs on any day after Day 3 in the presence of parasitaemia, without previously meeting any of the criteria of Early Treatment Failure;

(ii) Presence of parasitaemia and fever on any day after Day 3, without having previously meet the criteria of ETF.

Late Parasitological Failure (LPF):

Presence of parasitaemia on any day from day 7 onwards and axillary temperature <37.5 ºC, without previously meeting any of the criteria of Early Treatment Failure or Late Clinical

Failure.

*****The adequate clinical and parasitological response (ACPR)** is 1-TF. It is defined as absence of parasitaemia at the end of the follow up period (day 28), irrespective of axillary temperature without previously meeting any of the criteria of early and late treatment failure. In the adjusted estimates, patients with late asexual parasite reappearance (with or without fever) will be considered ACPR if the PCR analysis shows a new infection rather than a recrudescence.

- Secondary Endpoints

The secondary endpoints will be:

- Asexual parasite clearance time (PCT): Asexual parasite clearance time will be defined as the time (in days) from the day of randomization to 2 consecutive negative blood slides (collected at different days). The time to the event will be taken as the time to the first negative slide.
- Gametocytaemia (prevalence and density) at day 7, 14, 21, 28 after treatment;
- Hemoglobine changes between day 0 and day 14 and 28.

### Sample size calculation

Two different sample sizes were calculated: one for the estimation of *P.vivax* failure rate for chloroquine (28-day follow-up) and for Primaquine (2-year follow-up) in a cohort of *P.vivax* patients initially treated with the current radical cure regimen, and the second for the population survey on *P.vivax* prevalence (in a random sample of the study population).

- ***P.vivax population survey***

A total 2,100 individuals at risk of malaria were estimated in the 4 study communes (census February 2009). According to the provincial malaria station (PMS) data on surveys carried out in April-May, the overall parasite rate was around 16% (ranging from 5 to 39% across hamlets) and the prevalence of *P.vivax* at 9%. In order to detect a minimal prevalence of 9% with 3% precision and at 5% significance level, a total of 300 individuals is needed for the survey (“CSample” command/EpiInfo6). After “finite population correction” and adding 10% security margin, a final sample size of 300 individuals would be sufficient. Since there are a total of about 370 households and in order to simplify sampling procedures, one individual in each house visited for the screening will be randomly selected to participate in the survey. This will be done by randomly choosing from the census file. The selected individual will be asked oral informed consent before starting in-depth interview.

- ***P.vivax cohort***

Between 2003 and 2007, *P. vivax* late parasitological failure to CQ was monitored in several Central Vietnam provinces and ranged from 0% to 5.7% (13). Therefore, to detect a failure rate as low as 5% with a 3% precision at 5% significance level, a minimum of 185 individuals are needed (“CSample” command/EpiInfo6). Assuming a maximum drop out rate of 10% during the first 28 days, a total of 204 cases will be necessary. For the estimation of a similar PQ failure rate after 2 years of follow-up, a total of 230 patients should be included with a maximum of 20% drop out during the 2-year follow-up.

By including 250 vivax patients in the cohort, and assuming a maximum drop out rate of 20% during the 2-year follow-up, we can expect to get a final database of 200 patients with a complete follow-up (i.e. 31 successive sampling results) for the modelling of *P.vivax* new infections/relapses.

## Data collection

### Screening & survey

Prior to the start, a full census of the study population will be carried out with assignment of a unique code number to each individual living in the study area. The whole population will be asked to participate for a free clinical and biological examination for malaria parasites and adequate treatment when required. People will be asked an oral consent in the presence of the village leader and the hamlet health worker, and will be clinically examined by a medical doctor. Body temperature will be checked and a blood sample (finger prick) for microscopic examination (thick+thin film) will be collected. Microscopic examination will be done immediately: all non-P.vivax malaria infections (including all mixed infections) will be treated according to the national guideline, and other pathologies will be treated or referred accordingly. Patients with a *P.vivax* mono-infection, if eligible (see §selection criteria), will be invited to enter in the study cohort for a 2-year monthly follow up (written informed consent required). Non-eligible *P.vivax* patients (including those refusing consent), will still be offered the possibility to be treated free of charge according to the national treatment guidelines (CQ+PQ).

All data will be recorded onto a pre-coded standardised questionnaire (Annex1).

More detailed information on the socio-economic status, preventive measures employed and known malaria risk factors will be collected in one family member randomly chosen by drawing a folded paper containing a number (one number per household dwellers). The chosen number will correspond to the individual code number of the person to be included in the survey. If the chosen individual is absent or refuses to take part, he/she will not be replaced by another household member, the survey procedures will not be done in that household. Every effort will be made to trace an absent survey participant (survey team returning at different times during the whole duration of the survey in that village). After informed consent, the survey participant will undergo a detailed interview on his last months’ activities in relation to malaria exposure, and to preventive measures taken. In addition to the blood slide, one more blood sample will be taken for haemoglobin measurement (Hemocue) and another one on filter paper for later serology and genotyping. Microscopic examination will done on the spot and if a patient is detected with a *P.vivax* mono-infection, he will be invited to enter the cohort following similar procedures as during the screening (selection criteria and informed consent). Non eligible *P.vivax* patients, and other survey participants (with malaria or other diseases) will be treated according to the national guidelines.

All data will be recorded onto a pre-coded standardised questionnaire (Annex2).

### Passive case detection of *P.vivax* patients

Before the start of recruitement, all HHWs from the 4 study villages and the Tra Leng CHC staff will be trained to detect malaria patients with a rapid test, subsequently confirmed by microscopy reading, in order to complete the *P.vivax* patients’ recruitment. Symptomatic patients from the 4 study villages will be able to consult either their respective HHW or the CHC in Tra Leng for malaria diagnosis and treatment. At village level, malaria detection will be done with rapid diagnosis tests (RDT), secondarily confirmed by microscopic examination at the CHC before treatment administration. Patients will be first interviewed on malaria signs and treatments taken prior consulting, their body temperature checked, and blood samples for RDT and blood slide (thick+thin film) will be taken. Antipyretic treatment can be delivered before the slide results. If the patient presents with acute malaria symptoms and the RDT is positive for P.falciparum, he will be treated with Arterakin for 3 days following the national treatment guideline. If a patient is found positive with a P.vivax mono-infection, if eligible he will be invited to enter the cohort, after written informed, and will be treated and followed-up accordingly. Patients with mixed Pv infections or other malaria species will be treated according to the national guidelines (Arterakin 3 days if P.falciparum is present, CQ 3days for non Pf species) and other patients will be referred to the CHC if necessary. HHWs will only have the possibility to treat either malaria infections and/or simple viral infections treatment (antipyretic/painkiller). All data will be recorded onto a pre-coded standardised questionnaire (Annex9).

In addition to the PCD, monthly active detection of malaria cases was initiated in June 2009 in order to reinforce the recruitment process by PCD (low skills of HHWs). Once a month, each household is visited by the study team, and all febrile individuals get sampled (thick & thin film) in order to screen for malaria parasite. *Pvivax* mono-infecgtions are recruited into the cohort if eligible, Pf or mixed infections are treated according to the national guidelines.

### *P.vivax* patients’ treatment & cohort follow-up

Patients identified during either the initial screening/survey or during PCD and fulfilling all the inclusion criteria (all present) and exclusion (all absent) criteria will be included in the cohort. They will be treated with a 10-day radical cure regimen of Primaquine, and will be monitored during the first 28 days following standard WHO protocol for *in vivo* antimalaria drugs efficacy studies, then monthly checked for malaria symptoms and parasites for 23 months.

***Treatment of patients in the cohort***

The new treatment guideline for the radical cure of *P.vivax* (2007) consists of 25mg/kg Chloroquine (CQ) over 3 days, together with Primaquine (PQ), 0.5mg/kg daily for 10 days, under the direct supervision of the study team (Table 1). Patients will be observed during one hour: in case of vomiting within the first 30 minutes, a full dose will be re-administered, only half dose if vomiting occurs between 30 minutes and 1 hour. The total number of tablets of each drug administered (+/- re-administrated dose if vomiting) will be recorded on the patient’s follow-up form (Annex7)

**Table 1: Doses of CQ & PQ by body weight**

| **Weight (kg)** | **Primaquine base, 7.5 mg**  **Number of tablets/day**  **(Total = 10 days)** |
| --- | --- |
| **5-6** | 1/4 |
| **7-10** | 1/2 |
| **11-14** | 3/4 |
| **15-18** | 1 |
| **19-21** | 1+1/4 |
| **22-25** | 1+1/2 |
| **26-29** | 1+3/4 |
| **30-33** | 2 |
| **34-36** | 2+1/4 |
| **37-40** | 2+1/2 |
| **41-44** | 2+3/4 |
| **45-48** | 3 |
| **49-51** | 3+1/4 |
| **52-55** | 3+1/2 |
| **56-59** | 3+3/4 |
| **60+** | 4 |

| **Weight (kg)** | **Chloroquine base, 150mg**  **Number of tablets** | | |
| --- | --- | --- | --- |
|  | **Day0** | **Day 1** | **Day 2** |
| **5-6** | 1/2 | 1/4 | 1/4 |
| **7-10** | 1/2 | 1/2 | 1/2 |
| **11-14** | 1 | 1 | ½ |
| **15-18** | 1 | 1 | 1 |
| **19-24** | 1.5 | 1.5 | 1 |
| **25-35** | 2.5 | 2.5 | 1 |
| **36-50** | 3 | 3 | 2 |
| **50+** | 4 | 4 | 2 |

**28-day follow up**

During the 10-day treatment, patients will be examined daily, then weekly at day 14, 21 and 28, following detailed procedures in Table 2. Patients will be visited at home by the study team, and if the patient is absent every effort will be done to trace him before the next scheduled visit. Clinical signs and symptoms, especially adverse events (AE) will be assessed at each scheduled visit; early signs of haemolytic anaemia will be systematically checked (colour of urine and conjunctives). All signs and symptoms will be recorded on a standardised pre-coded questionnaire (Annex7). Blood samples will be collected according to the predefined schedule in Table 2. In case of fever, or any new symptoms appearing between the scheduled visits, patients will be asked to consult either their hamlet health worker (HHW) or the CHC for a clinical and biological assessment. In case of recurrent *P.vivax* parasitemia during the 28-day follow-up, a blood sample will be taken by finger prick (100µl with a heparinised µcapillary) for later dosage chloroquine blood level measurement. Any *P.vivax* treatment failure (early or late clinical failure), or a new infection with *P.falciparum,* will be treated with Arterakine for 3 days (Artesunate+piperaquine) according to the national guidelines. In this case, the patient will still remain in the follow-up (28-day and 2-year monthly follow-up).

**Table 2. Patients’ follow-up schedule during the first 28 days**

|  | **D0** | **D1** | **D2** | **D3** | **D4** | **D5** | **D6** | **D7** | **D8** | **D9** | **D14** | **D21** | **D28** | **Dx** | **M*** | **Mx** |
| --- | --- | --- | --- | --- | --- | --- | --- | --- | --- | --- | --- | --- | --- | --- | --- | --- |
| **PROCEDURES** |  |  |  |  |  |  |  |  |  |  |  |  |  |  |  |  |
| Clinical examination | **X** | **X** | **X** | **X** | **X** | **X** | **X** | **X** | **X** | **X** | **X** | **X** | **X** | **X** | **X** | **X** |
| Assess adverse event | **X** | **X** | **X** | **X** | **X** | **X** | **X** | **X** | **X** | **X** | **X** | **X** | **X** | **X** |  |  |
| Body temperature | **X** | **X** | **X** | **X** | **X** | **X** | **X** | **X** | **X** | **X** | **X** | **X** | **X** | **X** | **X** | **X** |
| Thick+thin film | **X** | **X** | **X** | **X** |  |  |  | **X** |  |  | **X** | **X** | **X** | **X** | **X** | **X** |
| Filter paper 2 spots  (ELISA & PCR) | **X** |  | **X** | **X** |  |  |  | **X** |  |  | **X** | **X** | **X** | **X** | **X** | **X** |
| Hemoglobin | **X** |  |  |  |  |  |  |  |  |  | **X** |  | **X** |  |  |  |
| Filter paper CQ  (100µl capillary) | **X** |  |  |  |  |  |  | **X** |  |  |  |  | **X** | **X** |  |  |
| Direct observation of treatment | **X** | **X** | **X** | **X** | **X** | **X** | **X** | **X** | **X** | **X** |  |  |  |  |  |  |

**Dx**: Any other day if the patient feels febrile or unwell

**M***: monthly visits by HHW from 2^nd^ to 24^th^ month

**Mx:** unscheduled monthly visit if patient feels sick

**Day 0: Enrollement/ administration of the study medication**

Demographic Data and Medical History

Demographic data and a general history of past and present illnesses will be recorded onto to the pre-coded standardised form for the 28-day follow-up.

Informed consent

After an in-depth interview, with one physician of the study or with another qualified person, the patient (or the parent/legal guardian) will be asked to document his/her consent by signing an informed consent form; if the patient is minor of age/not emancipated, the consent must be given by a parent or legal guardian according to national law. However, in this case, the investigator is responsible to check that the patient is also freely willing to take part in the study. The signed informed consent (or thumb-printed whenever the patient or the parents/guardians are illiterate) must be obtained before any tests or evaluations related to the study are carried out. However, a thick blood film or a rapid diagnostic test before the informed consent can be done as this can be considered a normal procedure for the management of patients suspected having clinical malaria.

Physical and Clinical Examination

A general physical examination will be performed.

A clinical examination will be performed: symptoms and axillary temperature (mercury thermometer).

Vital Signs and Weight

Vital signs (respiratory rate and pulse) and weight will also be measured (see details in patient’s form for 28-day follow-up).

Blood slides:

Thick and thin films will be prepared following the SOP (standard operating procedures) for laboratory. Blood slides will be examined for the confirmation of *P. vivax* mono-infection and parasite density.

Blood slides will be independently read by 2 qualified microscopists, data entered in separate files. Further details are described and explained in the SOP (SOP: Quality control (QC) guidelines for the laboratories participating in the study).

Parasite density will be calculated by counting the number of asexual parasites per 200 leukocytes in the thick blood film, based on an assumed WBC of 8,000 /µl by light microscopy at 1000xmagnification. The parasite density per microlitre will be calculated using the following formula:

Parasite density / µl = Number of parasites counted x 8,000

Number of leukocytes counted

PCR/ELISA

A blood sample (2 spots of at least 1.5cm diameter) will be collected on filter paper (Whatmann grade 3) at day 0 before treatment and every time a blood slide is done. Samples from patients classified as late treatment failure will be subsequently used for parasite genotyping. ELISA test for detecting CSP protein will be done on all blood samples at of the 28-day and monthly follow-up. Antibodies against the CSP are specifically produced during a new *P.vivax* infection (but not during relapses).

Haemoglobin (Hb)

Haemoglobin will be measured directly by using the HemoCue method following the SOP for laboratory. Briefly a drop of capillary blood is taken with a microcuvette which is then put in the reader. After 10” the Hb level appears on the screen. The result is directly recorded on the patient’s form.

Blood sample for CQ blood level measurement

100µl of capillary blood is taken with a microcapillary and poured onto a filter paper (Whatman grade 3).

Administration of the Study Drugs by a study doctor or nurse

CQ and PQ according to the patient’s body weight (Table 1).

Adverse Events Report

All adverse events will be recorded.

Concomitant Medications

Any medications taken by the study subject will be recorded.

**Day 1 -9: *Treatment Period***

Physical and Clinical Examination

A general physical examination and a clinical examination will be performed: symptoms, axillary temperature (mercury thermometer). An in-depth interview on potential risk factors (last month’s activities and malaria prevention measures taken) will be administered as soon as the patient feels well to answer questions. All data will be recorded on the patient’s follow-up (Annex7b)

Vital Signs

Vital signs (respiratory rate and pulse) will be measured.

Blood Slide at day 1-2-3 and 7

A thick and thin blood smear will be obtained to verify the presence and to determine the density of asexual and sexual stages of *P*. *vivax*.

PCR/ELISA

A blood sample will be collected any time a blood slide is done (Whatmann grade 3) for later genotyping.

CQ blood level at day7

100µl of capillary blood will be taken and poured on a filter paper (Whatmann grade 3) for later CQ blood level measurement in case of a *P.vivax* recurrence during the 28-day follow-up.

Concomitant Pharmacological Treatments

Concomitant medications being taken by the patients will be recorded.

Adverse Events Report

All adverse events will be recorded.

Administration of the Study Drugs by a study doctor or nurse

CQ at day 1 and 2. PQ from day 1 until day 9.

**Day 14 and 28: *Follow up***

Physical and Clinical Examination

A general physical examination and a clinical examination will be performed: symptoms, axillary temperature (electronic thermometer).

Vital Signs

Vital signs (respiratory rate and pulse) will be measured.

Blood Slide

A thick and thin blood smear will be obtained to verify the presence and to determine the density of asexual and sexual stages of *P*. *vivax*.

PCR/ELISA

A blood sample will be collected any time a blood slide is done (Whatmann grade 3) for later genotyping.

Haemoglobin

1 blood sample will be taken for direct Hb measurement (HemoCue).

Concomitant Pharmacological Treatments

Concomitant medications being taken by the patients will be recorded.

Adverse Events Report

All adverse events will be recorded.

**Day 21: *Follow up***

As for day 14, without Hb

**Unscheduled visit until day 28**

As Day 21. In case of a *P.vivax* recurrent infection, a blood sample for later CQ blood level measurement will be taken (100µl capillary blood onto a filter paper)

***2-year monthly follow-up***

After completion of the 28-day follow up, patients will be visited monthly at home by a health staff from the study team. Each patient will be interviewed on malaria signs/treatment since the last visit, examined clinically (body temperature) and a blood sample (slide +filter paper) will be taken. All information will be recorded on a pre-coded standardised questionnaire (monthly follow-up form). In case a patient presents with a recurrent *P.vivax* parasitemia s/he will be treated with CQ alone for 3 days. In case of persistent *P.vivax* gametocytemia, the patient can be treated with a single dose of Primaquine (0.75mg/kg). *P.falciparum* mono- or mixed infections will be treated with Arterakine according to the national guidelines. Blood slides will be stained and read within 48h at Tra leng CHC and stored as well as filter papers in a dry, cool place.

If the patient is not available on the day of the scheduled visit, it is the responsibility of the HHW to trace him/her back before the next scheduled visit. If a patient cannot be traced for a whole month, the HHW has to report it (together with the reason of absence) on the monthly follow-up form. Cohort patients will be asked to consult their HHW (or the CHC) for any symptom appearing between the monthly scheduled visits, for clinical and biological examination by the study team.

***Demographic surveillance of the study population***

Population movements will be monitored monthly via the HHWs who will report every month (monthly meeting with the study team) any change in the population in their respective cluster, i.e. newborns, immigrants, emigrants, deaths. Individual code numbers of emigrants and deaths will be frozen, while newborns and immigrants will receive a new individual code number. Within a registered family (already in the census) a new comer (newborn, immigrant) will receive an individual code number following the sequential numbering within that family, and his individual socio-demographic data should be completed on the family census form.

If a complete new household is set up in the village, it will be attributed a new house number following sequential house numbering in that village, and all family members will be attributed an individual code number following same procedures as during the census (01=household leader; 02=wife/husband; 03=first child, etc…). This new family should be interviewed to collect all socio-demographic data following the census form.

If an individual from a given household moves into another house within the study population (marriage, other reasons), he will keep his original code number. If a person moves out temporarily from the study area (code number frozen), and subsequently returns before the end of the study, he will keep his original individual code number and information on the reason and destination of his absence will be recorded in the census database.

## Blood samples processing

Blood slides will be examined directly, either in the field during the screening/survey or at Tra leng CHC during PCD and cohort follow-up. Thick and thin blood smears will be stained with Giemsa 4% for 45 minutes and examined for species diagnosis and parasite density. The later will be determined by counting the number of asexual parasites for 200 white blood cells (WBC) and assuming 8,000 WBC/µl. Slide reading will be blinded to the patient’s details and symptoms, first by the CHC microscopist, and systematically double checked by the senior microscopist from NIMPE. Discrepant results will be re-read by the 2 microscopists together. Quality control will be carried out, every 3 months, on all positive and 10% of negative slides at NIMPE, Hanoi.

Filter papers, after complete drying (outside direct sun light), will be kept in individual plastic bags, stored within a bigger bag containing silica gel (to avoid direct contact of blood with silicagel) and kept at 4°C (fridge) at Tra Leng CHC. CSP-ELISA and genotyping (by micro-satellites) will be carried at ITM Antwerp (Lab. Dept of Parasitology) by the Msc student from NIMPE, Hanoi during her 2^nd^ year of training, and after Msc graduation and transfer of techniques, at NIMPE, Hanoi.

- CSP-ELISA will be carried out on all survey samples to estimate the transmission intensity (anti-CSP antibodies prevalence), and on samples collected during 28-day and monthly follow up visits, to follow the evolution of anti-CSP Ab and identify possible new *P.vivax* infections. This technique is currently optimized and standardised by a Belgium PhD student, at ITM Antwerp.
- Species specific PCR will be performed on all blood samples collected on filter paper in order to confirm the *P.vivax* mono-infection (cohort patients), the overall species distribution and the prevalence of sub-patent malaria infections (survey samples). This will be done by using SnM-PCR^15^ (semi-nested multiplex PCR) to amplify ssrDNA sequences of Plasmodium species. Only samples with a *P. vivax* mono-infection at inclusion will be used for the analysis of the cohort patients.
- Molecular genotyping will be done using micro-satellites markers, a new technique currently set up and optimized at IMT, Antwerp laboratory. This PCR will be applied on all survey samples to characterize genetic diversity of *P.vivax* infections in the study area. For all patients included in the cohort, this technique will be applied each time a vivax parasitemia recurrence occurs during the 2-year follow-up, in order to the compare successive genotypes.

## Data entry & Analysis

All data will be double entered and cleaned in Epi-data, and statistical analyses will be carried out using STATA version 10. Regular backups (weekly) of the different databases will be done and copies will be kept at the provincial malaria station (PMS), at NIMPE, Hanoi, as well as ITM, Antwerp.

Allelic frequency, CSP-Ab prevalence (transmission intensity), malariometric indices, and malaria risk factors will be determined using all survey samples results from diagnostic PCR and genotyping, CSP-ELISA, slide reading, as well as epidemiological information. Moreover for the cohort database, each of treated *Pvivax* patients will have 31 successive sampling results over 2 years, with CSP and genotyping results for each vivax parasitaemia recurrence.

Together with the data on allele frequencies, transmission level and known risk factors for malaria infections, a mathematical model will be elaborated to calculate the probability for any *P.vivax* parasitemia to be a new infection. The mathematical model will be optimized in order to fit as much as possible the real data collected for the cohort patients.

# ETHICAL ISSUES

All patients in the four study villages will be offered free examination and treatment for malaria during the screening and population survey and based on their oral informed consent (in the presence of 2 community witnesses (hamlet leader and HHW)) will be enrolled. For the inclusion in the vivax cohort for a 2-year follow-up, all study procedures will be carefully explained to the eligible patients who will be recruited only after written informed consent. Patient information form (as well as assent form for minors) will be translated into Vietnamese language and, if necessary, orally translated into local (M’nong and Cadong) language during the survey/ACD and PCD.

It will be made clear by the study team to the whole study population and especially to hamlet leaders and community representatives that study participation will be strictly on a voluntary basis. People refusing to participate will by no means be disadvantaged or harmed in terms of general health care services and can benefit from adequate treatment for *P.vivax* infection even without being included in the study cohort.

# REFERENCES

1. NIMPE. Annual Report of the National Malaria Control Program in Vietnam: 2003. NIMPE. 2004. Hanoi, Vietnam.
2. NIMPE. Annual Report of the National Malaria Control Program in Vietnam: 2004. NIMPE. 2005. Hanoi, Vietnam.
3. Erhart A, Thang ND, Hung NQ, Toi LV, Hung LX, Tuy TQ, et al. Forest malaria in Vietnam: a challenge for control. Am.J.Trop.Med.Hyg. 2004; 70:110-118.
4. Erhart A, Ngo DT, Pham VK, Ta TT, Van Overmeir C, Speybroeck N, et al. Epidemiology of forest malaria in Central Vietnam: a large scale cross-sectional survey. Malaria Journal 2005, 4:58 (<http://malariajournal.com/content/4/1/58>)
5. WHO. 2006. Guidelines for treatment of malaria. WH0 Press. Geneva, Switzerland.
6. Ministry of Health. Guideline for malaria treatment. Hanoi, Vietnam. 1992
7. De Vries. An individual orientated approach to modelling demography malaria and illness. http://www.demogr.mpg.de/Papers/workshops/010221_paper05.pdf
8. Imwong M, Snounou G, Pukrittayakamee S, Tanomsing N, Kim JR, Nandy A, et al. Relapses of *Plasmodium vivax* infection usually result from activation of heterologous hypnozoites. J.Infect.Dis. 2007; 195: 927-933.
9. Suphavilai C, Looareesuwan S, Good MF: Analysis of circumsporozoite protein-specific immune responses following recent infection with *Plasmodium vivax***.** *Am J Trop Med Hyg* 2004, **71:**29-39.
10. Herrera S, Bonelo A, Perlaza BL, Valencia AZ, Cifuentes C, Hurtado S, et al. Use of long synthetic peptides to study the antigenicity and immunogenicity of the *Plasmodium vivax* circumsporozoite protein. Intern.J.Parasitol.2004; 34:1535-1546
11. Dr Nguyen Van Van. Malaria Review in Quang Nam Province: 1976-2006. Provincial Malaria Station, 2007. Internal Report. Tam Ky, Vietnam.
12. Annual report on malaria entomological collections in Quang Nam province: 2007. Provincial Malaria Station, 2008. Internal Report. Tam Ky, Vietnam.
13. Nong Thi Tien. Reviewing the monitoring of antimalarial drug efficacy and resistance in sentinel sites in Vietnam. International Colloquium on Malaria control in the Mekong Region: challenges and opportunities. Hanoi, Dec.2007. Abstract book p.73. Edited by Hanoi Medical Publishing House.
14. Rubio JM, Benito A, Berzosa PJ, Roche J, Puente S, Subirats M, et al. Usefulness of seminested multiplex PCR in surveillance of imported malaria in Spain. J.Clin.Microbiol. 1999;37:3260-4.
15. Drakeley CJ, Corran PH, Coleman PG, Tongren JE, McDonald SLR, Carneiro I, et al. Estimating medium and long term trends in malaria transmission by using serological markers of malaria exposure. Proc.Nat.Acad.Sciences 2005; 102 (14):5108-5113.
16. Berkvens D, Speybroeck N, Praet N, Adel A, Lesaffre E. Estimating disease prevalence in a Bayesian framework using probabilistic constraints. Epidemiology 2006; 17(2): 145-153.

**ANNEX A: Geographical situation of the study site in Vietnam**


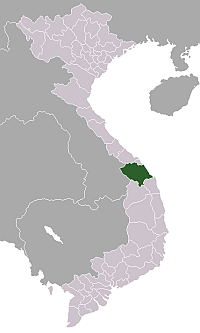

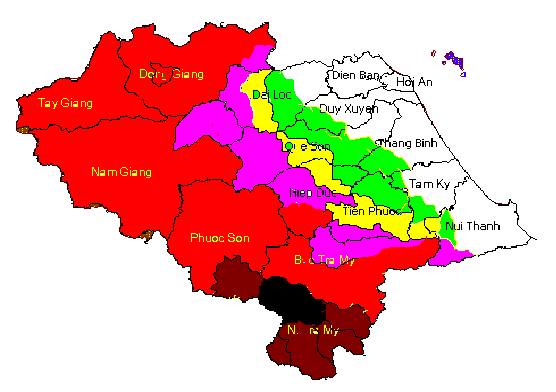


**Hué**

**Sea**

**Laoss**

**Laos**

**Quang Nam province: malaria stratification**

**South**

**Tra My**

**Map of Vietnam**

**ANNEX B. DEFINITION OF SEVERE FALCIPARUM MALARIA***

**General danger signs of severe illness in children**

- Inability to drink (or breastfeed)
- Repeated vomiting
- Recent history of convulsions
- Lethargy or unconsciousness
- Inability to sit or stand up

**Severe manifestations of *P.falciparum* malaria** in adults and children

| **Prognostic value** | |  | **Frequency** | |
| --- | --- | --- | --- | --- |
| Children | Adults |  | Children | Adults |
| *Clinical manifestations* | | | | |
| + | ?^b^ | Prostration | +++ | +++ |
| +++ | + | Impaired consciousness | +++ | ++ |
| +++ | +++ | Respiratory distress (acidotic breathing) | +++ | + |
| + | ++ | Multiple convulsions | +++ | + |
| +++ | +++ | Circulatory collapse | + | + |
| +++ | +++ | Pulmonary oedema (radiological) | +/- | + |
| +++ | ++ | Abnormal bleeding | +/- | + |
| ++ | + | Jaundice | + | +++ |
| + | + | Haemoglobinurea | +/- | + |
| *Laboratory findings* | | | | |
| + | + | Severe anaemia | +++ | + |
| +++ | +++ | Hypoglycaemia | +++ | ++ |
| +++ | +++ | Acidosis | +++ | ++ |
| +++ | +++ | Hyperlactataemia | +++ | ++ |
| +/- | ++ | Hyperparasitaemia | ++ | + |
| ++ | ++ | Renal impairment | + | +++ |

^a^ On a scale from + to +++: +/- indicates infrequent occurrence. ^b^ Data not available

(*) WHO. Severe falciparum malaria. Transactions of the Royal Society of Tropical Medicine and Hygiene, 2000, 94(Suppl.1): 1-90.

**ANNEX C. DRUG INTERACTIONS**

**DRUGS TO BE AVOIDED DURING PRIMAQUINE TREATMENT (interactions)**

- **DAPSONE:** leprosy, skin infections (Dapsone, DDS)
- **SULFONAMIDES:** antibiotic for bronchitis, urinary infections, diarrheas (Bactrim, Cotrim, Sulfatrim,…)
- **SULFOXONE:** sulfonamide for leprosy, dermatitis herpetiformis (Aldapsone, DDF, Diazone)
- **METHYLDOPA**: anti-hypertensive (Aldochlor, Aldoril)
- **FURAZOLIDONE:** infectious diarrhea and enteritis (Furoxone)
- **NITROFURANTOINE:** urinary tract infections (Furadantin)
- **QUINACRINE:** anti-malaria, antibiotic for Giardiasis (Atabrine)
- **QUINIDINE:** anti-arythmic (Cardioquin, Quinora , Quinidine SR …)
- **QUININE**: antimalarial (Quinine sulfate, Quinimax, etc…)
- **VITAMINE K:** prevention of bleeding in newborns (Phytonadione)

**ANTIBIOTICS WITH ANTIMALARIAL EFFECT (Avoid during 28-day follow-up)**

- **Doxycycline**
- **Erythromycine**
- **Azythromycine**
- **Trimethoprim**
- **Clindamycine**
- **Rifampicin**
- **Pentamidine**
