# Supplementary material for: Plasmodium vivax morbidity after radical cure: A cohort study in Central Vietnam
Source: PLoS Med. 2019 May 17;16(5):e1002784. doi: 10.1371/journal.pmed.1002784 (PMC6524795; doi:10.1371/journal.pmed.1002784)
Supplement: S2 Text — STROBE Statement is a checklist of 22 items that we consider essential for good reporting of cohort studies. These items relate to the article′s title and abstract (item 1), the introduction (items 2 and 3), methods (items 4–12), results (items 13–17) and discussion sections (items 18–21), and other information (item 22 on funding). STROBE, Strengthening The Reporting of OBservational Studies in Epidemiology. (DOC) [file pmed.1002784.s007.doc]

STROBE Statement—Checklist of items that should be included in reports of ***cohort studies***

|  | Item No | Recommendation |
| --- | --- | --- |
| **Title and abstract** | 1 | (*a*) Indicate the study’s design with a commonly used term in the title or the abstract  *DONE- described in the title as “cohort study”* |
|  |  | (*b*) Provide in the abstract an informative and balanced summary of what was done and what was found  DONE-we  *- Describe the main analysis performed: “Time to first vivax recurrence was estimated by Kaplan Meier survival analysis, and risk factors for first- and recurrent infections identified by Cox regression models”*  *- Describe what was found: “Most individuals (76.78%, 171/223) had recurrent vivax infections identified by molecular methods (PCR); in about half of them (55.61%, 124/223) infection was detected by microscopy, and 84 individuals (37.67%) had symptomatic recurrences. Median time to 1^st^ recurrence by PCR was 118 days (IQR [59; 208]). The estimated probability of remaining free of recurrence by month 24 was 20.40% (95%CI [14.42; 27.13]) by PCR, 42.52% (95%CI [35.41; 49.44]) by microscopy, and 60.69% (95%CI [53.51; 67.11]) for symptomatic recurrences. The main risk factor for recurrence (first- or recurrent-) was prior P. falciparum infection.”* |
| Introduction | | |
| Background/rationale | 2 | Explain the scientific background and rationale for the investigation being reported  *DONE- in paragraph 1 we describe the public health context of theP. vivax radical cure treatment, paragraph 2, describes P. vivax burden in Vietnam, paragraph 3 gives current malaria control programme in Vietnam. Paragraph 4 identifies challenging of PQ treatment P. vivax in Vietnam and paragraph 5 builds the rationale for our study.* |
| Objectives | 3 | State specific objectives, including any prespecified hypotheses  *DONE – In the last paragraph of the introduction, we state:”* *We report here results of a cohort study where P. vivax patients were treated radically with the previously recommended high dose PQ regimen (0.50mg/kg/day for 10 days) and followed up monthly for up to 2 years, with the aim of evaluating the post-treatment transmission dynamics of recurrent vivax infections”* |
| Methods | | |
| Study design | 4 | Present key elements of study design early in the paper  *DONE – In the first paragraph of the study design, we state the key features of the study: “This is a 2-year prospective cohort study carried out in Central Vietnam in which P. vivax infected patients were followed up monthly after radical treatment”* |
| Setting | 5 | Describe the setting, locations, and relevant dates, including periods of recruitment, exposure, follow-up, and data collection  *DONE – In three paragraphs of Study site and population, Study design and Data collection are under the Methods section.* |
| Participants | 6 | (a) Give the eligibility criteria, and the sources and methods of selection of participants. Describe methods of follow-up.  *DONE – In the paragraph of Data collection is under the Methods section.* |
|  |  | (b) For matched studies, give matching criteria and number of exposed and unexposed  *This study is not a matched study.* |
| Variables | 7 | Clearly define all outcomes, exposures, predictors, potential confounders, and effect modifiers. Give diagnostic criteria, if applicable  *DONE- in the paragraph of Definition is under the Methods section.* |
| Data sources/ measurement | 8* | For each variable of interest, give sources of data and details of methods of assessment (measurement). Describe comparability of assessment methods if there is more than one group  *DONE- in six paragraphs of Data analysis under methods section.* |
| Bias | 9 | Describe any efforts to address potential sources of bias |
| Study size | 10 | Explain how the study size was arrived at  *DONE- in paragraph of Sample sizes under methods section.* |
| Quantitative variables | 11 | Explain how quantitative variables were handled in the analyses. If applicable, describe which groupings were chosen and why  *DONE- in six paragraphs of Data analysis under methods section.* |
| Statistical methods | 12 | (a) Describe all statistical methods, including those used to control for confounding  *DONE- in six paragraphs of Data analysis under methods section.* |
|  |  | (b) Describe any methods used to examine subgroups and interactions  *DONE- in six paragraphs of Data analysis under methods section.* |
|  |  | (c) Explain how missing data were addressed  *DONE- in six paragraphs of Data analysis under methods section.* |
|  |  | (d) If applicable, explain how loss to follow-up was addressed  *DONE- in six paragraphs of Data analysis under methods section.* |
|  |  | (e) Describe any sensitivity analyses  *DONE- in six paragraphs of Data analysis under methods section.* |
| Results | | |
| Participants | 13* | (a) Report numbers of individuals at each stage of study—eg numbers potentially eligible, examined for eligibility, confirmed eligible, included in the study, completing follow-up, and analysed  *DONE-in two paragraphs of Cohort characteristics under Results section.* |
|  |  | (b) Give reasons for non-participation at each stage  *DONE-in two paragraphs of Cohort characteristics under Results section.* |
|  |  | (c) Consider use of a flow diagram  *DONE-Fig 1* |
| Descriptive data | 14* | (a) Give characteristics of study participants (eg demographic, clinical, social) and information on exposures and potential confounders  *DONE-in two paragraphs of Cohort characteristics under Results section.* |
|  |  | (b) Indicate number of participants with missing data for each variable of interest  *DONE-in two paragraphs of Cohort characteristics under Results section.* |
|  |  | (c) Summarise follow-up time (eg, average and total amount)  *DONE-in two paragraphs of Cohort characteristics under Results section.* |
| Outcome data | 15* | Report numbers of outcome events or summary measures over time  *DONE-in two paragraphs of Characteristics of vivax recurrences under Results section.* |
| Main results | 16 | (a) Give unadjusted estimates and, if applicable, confounder-adjusted estimates and their precision (eg, 95% confidence interval). Make clear which confounders were adjusted for and why they were included  *DONE- in two paragraphs of Probability of remaining free of vivax recurrence and four paragraphs of Risk factors for P. vivax recurrences under Results section.* |
|  |  | (b) Report category boundaries when continuous variables were categorized  *DONE- in two paragraphs of Probability of remaining free of vivax recurrence and four paragraphs of Risk factors for P. vivax recurrences under Results section.* |
|  |  | (c) If relevant, consider translating estimates of relative risk into absolute risk for a meaningful time period  *DONE- in two paragraphs of Probability of remaining free of vivax recurrence and four paragraphs of Risk factors for P. vivax recurrences under Results section.* |
| Other analyses | 17 | Report other analyses done—eg analyses of subgroups and interactions, and sensitivity analyses  *DONE- in four paragraphs of Risk factors for P. vivax recurrences under Results section.* |
| Discussion | | |
| Key results | 18 | Summarise key results with reference to study objectives  *DONE- summarised in first paragraph of Discussion section.* |
| Limitations | 19 | Discuss limitations of the study, taking into account sources of potential bias or imprecision. Discuss both direction and magnitude of any potential bias  *DONE-in paragraphs 7, 8 and 9 under Discussion section.* |
| Interpretation | 20 | Give a cautious overall interpretation of results considering objectives, limitations, multiplicity of analyses, results from similar studies, and other relevant evidence  *DONE-in paragraphs 2, 3, 4, 5, 6 and 7 under Discussion section.* |
| Generalisability | 21 | Discuss the generalisability (external validity) of the study results  *DONE-in paragraphs 2, 3, 4, 5, 6 and 7 under Discussion section.* |
| Other information | | |
| Funding | 22 | Give the source of funding and the role of the funders for the present study and, if applicable, for the original study on which the present article is based  *DONE-The present article is based not according to PLOS Med policy.* |

*Give information separately for exposed and unexposed groups.

**Note:** An Explanation and Elaboration article discusses each checklist item and gives methodological background and published examples of transparent reporting. The STROBE checklist is best used in conjunction with this article (freely available on the Web sites of PLoS Medicine at http://www.plosmedicine.org/, Annals of Internal Medicine at http://www.annals.org/, and Epidemiology at http://www.epidem.com/). Information on the STROBE Initiative is available at http://www.strobe-statement.org.
